# Impaired signaling for neuromuscular synaptic maintenance is a feature of Motor Neuron Disease

**DOI:** 10.1186/s40478-022-01360-5

**Published:** 2022-04-25

**Authors:** Qiao Ding, Kaamini Kesavan, Kah Meng Lee, Elyse Wimberger, Thomas Robertson, Melinder Gill, Dominique Power, Jeryn Chang, Atefeh T. Fard, Jessica C. Mar, Robert D. Henderson, Susan Heggie, Pamela A. McCombe, Rosalind L. Jeffree, Michael J. Colditz, Massimo A. Hilliard, Dominic C. H. Ng, Frederik J. Steyn, William D. Phillips, Ernst J. Wolvetang, Shyuan T. Ngo, Peter G. Noakes

**Affiliations:** 1 School of Biomedical Sciences, The University of Queensland, St. Lucia, QLD 4072 Australia; 2 Pathology Queensland, Faculty of Medicine, Royal Brisbane and Women’s Hospital, Herston, QLD 4006 Australia; 3 Australian Institute for Bioengineering and Nanotechnology, The University of Queensland, St. Lucia, QLD 4072 Australia; 4 Department of Neurology, Royal Brisbane and Women’s Hospital, 4006, Herston, QLD Australia; 5 University of Queensland Centre for Clinical Research, The University of Queensland, Herston, QLD 4006 Australia; 6 Department of Neurosurgery, Royal Brisbane and Women’s Hospital, Herston, QLD 4006 Australia; 7 Clem Jones Centre for Ageing Dementia Research, Queensland Brain Institute, The University of Queensland, St. Lucia, QLD 4072 Australia

**Keywords:** Amyotrophic Lateral Sclerosis, ALS, Neuromuscular junction, MuSK, Agrin, Motor neurons, Acetylcholine receptors

## Abstract

A central event in the pathogenesis of motor neuron disease (MND) is the loss of neuromuscular junctions (NMJs), yet the mechanisms that lead to this event in MND remain to be fully elucidated. Maintenance of the NMJ relies upon neural agrin (*n*-agrin) which, when released from the nerve terminal, activates the postsynaptic Muscle Specific Kinase (MuSK) signaling complex to stabilize clusters of acetylcholine receptors. Here, we report that muscle from MND patients has an increased proportion of slow fibers and muscle fibers with smaller diameter. Muscle cells cultured from MND biopsies failed to form large clusters of acetylcholine receptors in response to either non-MND human motor axons or *n*-agrin. Furthermore, levels of expression of MuSK, and MuSK-complex components: LRP4, Caveolin-3, and Dok7 differed between muscle cells cultured from MND patients compared to those from non-MND controls. To our knowledge, this is the first time a fault in the *n*-agrin-LRP4-MuSK signaling pathway has been identified in muscle from MND patients. Our results highlight the *n*-agrin-LRP4-MuSK signaling pathway as a potential therapeutic target to prolong muscle function in MND.

**Supplementary Information:**

The online version contains supplementary material available at 10.1186/s40478-022-01360-5.

## Introduction

Motor Neuron Disease (MND) is a devastating disorder with death occurring in approximately 80% of patients within 3–5 years of symptom onset [1]. A key feature of lower motor neuron involvement in MND is denervation, due to the withdrawal of motor nerve terminals from their target muscle cells [2–6]. Progressive denervation leads to progressive muscle weakness and wasting. Our limited understanding of the processes leading to loss of neuromuscular synapses in MND contributes to the lack of an effective treatment [5, 7]. Much is known of the mechanisms that maintain NMJs in health. Here, we report that these mechanisms might fail in MND, and the role of muscle in MND pathology.

We and others have shown that neural-agrin (*n*-agrin), a heparin sulphate proteoglycan released by the motor nerve terminal [8], plays an integral role in the stability of neuromuscular synapses [9–11]. *N*-agrin binds to the LRP4-MuSK receptor complex in the postsynaptic muscle membrane, activating a tyrosine kinase signaling cascade that stabilizes clusters of acetylcholine receptors (AChRs) in the muscle membrane [12, 13]. Upon initial phosphorylation of MuSK, dimers of Dok7 are recruited to the cytoplasmic region of MuSK to enhance its autophosphorylation [14, 15]. Caveolin-3 also binds to MuSK and is thought to aid in the phosphorylation process [16]. Activation of MuSK drives activation of Abl and Src kinases to phosphorylate the AChR-β subunit [10]. Rapsyn, a cytoplasmic scaffolding protein, is then recruited to stabilize AChRs in large postsynaptic clusters that mediate neuromuscular transmission [10, 11].

Impairment of the postsynaptic *n*-agrin-LRP4-MuSK signaling pathway can lead to failure and loss of the neuromuscular junction (NMJ). For example, mutations of agrin, LRP4, MuSK, Dok7 and rapsyn can each cause severe weakness by hampering the maturation and function of NMJs [17–21]. Autoantibodies targeting MuSK and LRP4 can disrupt *n*-agrin-MuSK signaling in adults, causing disassembly of the mature NMJ [22–24]. In the SOD1^G93A^ mouse model of MND, others have reported altered immunostaining for LRP4 and rapsyn [25]. Whether the abnormalities observed in MND mice are secondary to pathology in the nerve fiber, or whether they originate with postsynaptic defects in muscle remains unknown [3, 7, 26].

Several groups have reported disassembly of the NMJ, with evidence of nerve terminal withdrawal, flattening of the postsynaptic membrane, and localized axonal sprouting from individuals in the early stages of MND [2, 4, 26, 27]. In the present study, we report perturbed localization and expression of MuSK at the motor endplate and failure of muscles cells cultured from MND patients to respond properly to motor nerves or *n*-agrin. Our observations indicate that abnormalities in postsynaptic *n*-agrin-LRP4-MuSK signaling could contribute to muscle weakness and disease progression in MND and highlight muscle as an additional target for new therapeutic approaches in MND [6, 28, 29].

## Methods

### Study population

Research participants were recruited from the Royal Brisbane & Women’s Hospital (RBWH) MND clinic. Inclusion criteria for patients with MND were probable diagnosis of sporadic or familial MND, according to the El Escorial criteria [30]. For surgical controls, inclusion was based on symptoms of a neuromuscular disorder other than MND, requiring a muscle biopsy for diagnosis, as well as being between 25 to 80 years. Their final diagnosis is listed in Table 1. Healthy controls who provided needle punch biopsies were recruited as a convenience sample of family and friends of patients with MND. Exclusion criteria were as follows: aged less than 18 years, pregnancy, standard contradictions to undergoing a muscle biopsy, and unable to give informed consent due to cognitive impairment.

**Table 1:** Open Muscle biopsies subjected to pathological analyses, and generation of muscle satellite cells for in vitro studies

Donor IDs	Sex	Age	Diagnosis	Site of onset	ALSFRS-R (at biopsy)	Interval—Symptom Onset to Biopsy months	No NMJs imaged	Muscle pathology
Con-3#	M	44					19^$^	Normal
Con-4	M	54					22^$^, 7^*^	Inclusion Body Myositis
Con-5	M	39					12^$^	Focal Denervation
MND-1^	M	57	Sporadic MND	Spinal	34	10	4*	Chronic progressive denervation, early fiber type grouping
MND-3^	F	56	Sporadic MND	Spinal	N/A	10	40^$^, 5^*^	Chronic progressive denervation, early fiber type grouping
MND-4	M	61	Sporadic MND	Spinal	41	60	24^$^	Chronic denervation; collateral reinnervation, fiber type grouping
MND-5	M	63	Sporadic MND	Spinal	40	31	2*	Chronic progressive denervation, fiber type grouping
MND-6	F	77	Sporadic MND	Bulbar	29	31	3*	Chronic progressive denervation, fiber type grouping
MND-7^	F	68	Sporadic MND	Spinal	40	12		Denervation atrophy, early fiber type grouping

Con = Control (non-MND other disease control); ALSFRS-R—ALS Functional Rating Scale Revised; ^#^Deltoid muscle biopsy, the reminder are biopsies from *Vastus Lateralis* muscle; ^$^NMJs found in whole mount intact muscle fiber samples; *NMJs found in cryosections used in locating MuSK at the NMJ; ^Muscle also used to derive muscle satellite cells for in vitro studies

### Muscle biopsy and culture

#### Surgical biopsy

Biopsy procedures were overseen by neurosurgeon Dr Jeffree, and neurologists Drs Henderson and McCombe. Biopsies were taken from the *vastus lateralis* muscle or from the *deltoid* muscle of each participant. Muscle specimens were collected by RBWH neurosurgeons employing standard sterile conditions for the performance of a muscle biopsy, using the motor point method to collect the specimen from the area of the muscle with the highest density of NMJs [31], surgical details are provided in supplementary methods (Additional file 1: Online resource 1). Each biopsy specimen was divided into two samples: one for pathological assessment for diagnosis, and one for research. Pathological specimens were sent to Pathology Queensland by the attending neurosurgeon. Research samples were transferred into a 50 mL conical centrifuge tube containing Dulbecco’s Modified Eagle Medium/Ham’s F-12 (DMEM/F-12) with 0.5% gentamicin (ThermoFisher, MA, USA), and placed on ice for transport to our laboratory. Demographics for participants who provided surgical biopsy samples are presented in Table 1. Needle punch muscle biopsies were obtained from MND and control participants by a qualified general practitioner. Muscle biopsies were collected from the *vastus lateralis* of one leg and placed into transport media (described above; see supplementary methods for details (Additional file 1: Online resource 1)). Demographics for participants who provided needle punch biopsy samples are presented in Table 2.

**Table 2:** Muscle punch biopsies subjected to pathological analyses, and for generation of muscle satellite cells for in vitro studies

Donor IDs	Sex	Age	Diagnosis	Mutation (if known)	Site of onset	ALSFRS-R (at biopsy)	No NMJs	Interval—Symptom Onset to Biopsy
Con-1	F	54					2^$^, 7^*^	
Con-2	M	54					12^$^, 2^*^	
Con-6	F	65						
Con-7	M	65						
Con-8	M	47						
Con-9	M	61						
Con-10	M	71						
Con-11	F	65						
Con-12	M	70						
Con-13	M	44						
Con-14	M	57						
Con-15	M	32						
Con-16	M	64						
MND-2	M	47	Sporadic		Spinal	42	13^$^	7.8
MND-8	M	65	Sporadic		Spinal	25		2.9
MND-9	M	51	Familial	SOD1	Spinal	40		6.2
MND-10	M	47	Familial	C9orf72	Spinal	41		10.2
MND-11	M	49	Sporadic		Spinal	40		5.5
MND-12	M	54	Sporadic		Spinal	33		57.0
MND-13	M	53	Sporadic		Spinal	33		20.4
MND-14	F	58	Sporadic		Spinal	35		33.5
MND-15	M	79	Sporadic		Spinal	28		57.1
MND-16	M	46	Familial	C9orf72	Bulbar/Spinal	41		15.9

Con = healthy donor control (Non-MND); ALSFRS-R—ALS Functional Rating Scale Revised; ^$^Indicates NMJs found in intact muscle fiber samples; *Indicates NMJs found in cryosections

Muscle satellite (stem) cells were isolated from surgical and needle biopsies using an explant culture approach [32], and frozen as low passage cell stocks. The procedure was performed by the one person (Ms White), who was blinded to MND status. These myogenic cells were maintained in growth media consisting of DMEM/F-12 medium supplemented with 20% foetal bovine serum (FBS), 10% AmnioMAX C-100 and 0.5% gentamicin. Myogenic cultures received fresh media every second day and cells were sub-cultured at 70% confluence. When muscle satellite (stem) cells were plated out for experiments, cultures were maintained in growth media until reaching 70–80% confluence. At this stage, these myogenic cells were switched into differentiation media (DMEM-F12; 2% non-heat inactivated horse serum; 0.5% gentamycin (ThermoFisher)) to induce the formation of myoblasts and their subsequent fusion into multinucleated myotubes.

### Muscle pathology

Assessment of pathology of surgical muscle samples was conducted by Pathology Queensland. Muscle samples were frozen, cryo-sectioned transversally at ~ 8 µm [33], and fixed for 5 min in 4% PFA in PBS. They were then processed for hematoxylin and eosin (H&E) staining, and fiber typing immunohistochemistry (slow myosin and fast myosin), as per [34]. In brief, muscle sections were double stained with anti-MHC fast (Leica WB-MHCf) and anti-MHC slow (Lecia WB-MHCs), probed for with enzyme based secondary antibodies (i.e. peroxidase reacted 2nd antibody brown stain fast MHC fibers, and alkaline phosphatase reacted secondary antibody red (Leica-BOND Polymer Refine Red Detection system) or purple stained slow MHC fibers).

### Electron microscopy

At the time of muscle collection, a 1 mm diameter bundle of muscle fibers was teased away from the biopsy, and immersion fixed in 2.5% glutaraldehyde plus 4% PFA in PBS for at least 12 h. These samples were then processed for transmission electron microscopy, as per [35, 36]. Muscle sections were viewed and imaged using a JEOL 1010 and/or a Hitachi HT7700 transmission electron microscope.

### H9-hESC motor neuron differentiation

The H9 human embryonic stem cell line (H9 hESC, WA09; [37]) was obtained from WiCell Research Institute—National Stem Cell Bank, USA (RRID CVCL 9973). These cells were stably transfected with a GcaMP6f expression construct (H9-GcaMP6f) by StemCore at the Australian Institute for Bioengineering and Nanotechnology, UQ. Both H9-GcaMP6f and H9 (WA09) embryonic stem cells were grown on Matrigel-coated plates in conditioned media and dissected into small pieces (~ 200 μm^3^). This was considered as day 0. Following the protocol established by Du and colleagues [38], these cells were grown and dissociated, and allowed to differentiate for a further 10 days (see supplementary methods (Additional file 1: online resource 1)*.* At day 29, the neurons were assessed for viability and function, followed by RNA extraction, or fixing for immunostaining.

### Calcium imaging in GCaMP6f derived MNs

The H9-GCaMP6f embryonic stem cells were differentiated into motor neurons as described above. On day 29 of differentiation, neurons were washed twice with Hank’s Balanced Salt Solution (HBSS; ThermoFisher), after which they were bathed in HBSS. Cells were depolarized by adding KCl (50 mM) to the HBSS. The resulting green fluorescence was immediately recorded using a Leica DMi8 Inverted High Content Imager. The quantification of the emitted fluorescence employed Imaris image software (Bitplane, Oxford Instruments, UK). The fluorescence intensity at each time point after the addition of KCl was calculated using the following formula, ΔF/F_0_ = (F − F_0_)/F_0_ where F represents the fluorescence intensity at each time point subtracted from the Fluorescence intensity at time zero F_0_ [39].

### Quantitative Real-Time Polymerase Chain Reaction (qPCR)

Total RNA was isolated from H9 hESCs using a MACHEREY–NAGEL RNA extraction kit, and a NucleoSpin RNA Midi protocol (Macherey–Nagel GmbH & Co. KG, Germany, 740955.250). Total RNA (500 ng per sample) was used for cDNA synthesis, using the iScript cDNA synthesis kit (Bio-Rad, NSW Australia, 170–8890). The qPCR reaction was set up by adding SsoFast SsoFast EvaGreen supermix 5 µl (Bio-Rad, 1725200), 2 µl of cDNA, and 0.2 µl of forward primer and reverse primer. The sequences of the forward and reverse primers and their target gene product size are shown in Additional file 1: Table S3 (Additional file 1: online resource 2). This PCR mixture was run at 95 °C for 1 min, 95 °C for 7 s, 60 °C for 15 s, 65 °C for 5 s, 95 °C for 0.5 s for 40 cycles.

### Immunostaining of H9 motor neurons

H9 derived motor neurons (H9-MNs) were fixed with 4% PFA in PBS for 10 min at room temperature, washed 3 times in PBS, and then incubated in blocking buffer. They were then incubated overnight with primary antibodies to Islet1 and ChAT at 4 °C. Cells were washed 3 × 5 min with PBS prior to incubation with the appropriate Alexa-conjugated second antibody: Alexa-488 donkey anti-goat, Alexa-555 sheep anti-rabbit for 1 h at room temperature. Cover slips containing H9-MNs were then washed 3 × 5 min with PBS and mounted with Prolong Gold anti-fade medium containing DAPI (Invitrogen) onto glass slides. Antibody details are listed in Additional file 1: Table S4 (Additional file 1: online resource 2).

### Microfluidic cultures of human derived MNs and human muscle

H9 hESCs were differentiated into motor neurons as described above. At day 19 of differentiation, these committed motor neurons were plated in chamber one of a two-chamber microfluidic device (Merck, AX45005PBC). They were allowed to develop in chamber one for approximately two weeks (day 0 to day 14) sending their axons out along the connecting microgrooves towards chamber 2. When the motor axons reached the edge of channel 2, muscle satellite cells (50,000 cells) were plated into chamber 2 of the microfluidic device. At 80% confluence, myoblasts were switched into differentiation medium to induce the formation of multinucleated myotubes. The co-culture was grown for a further 2 weeks (day 14 to day 30), and medium was changed every 2 days. At day 30, chamber 2 was fixed and immunostained as described below. For immunostaining of cultures, AChRs were located with Alexa-488 α-bungarotoxin (Invitrogen), and motor axons were stained for neurofilament and SV2 and located with appropriate second antibodies (details in supplementary methods (Additional file 1: online resource 1). Antibody details are listed in Additional file 1: Table S4 (Additional file 1: online resource 2). Image acquisition of H9-MNs, and H9-MN muscle microfluidic cultures are given in supplementary methods (Additional file 1: online resource 1).

### Isolation of single nuclei from myotubes for RNA sequencing

Primary myogenic cells were seeded at a density of 1 × 10^5^ cells per 10 cm (0.01% gelatin coated) dish containing 3 mL of growth medium. Growth media was replaced every 48 h. At 70–80% confluence, growth medium was substituted for differentiation medium. At day 6 of myotube differentiation, single nuclei were isolated from myotube cultures for single nuclei RNA-sequencing using a protocol optimized from ([40]; detailed in supplementary methods, see Additional file 1: online resource 1). In brief, nuclei were extracted with cold nuclear extraction buffer, resuspended, and passed through 70 and 40 mm filters. Nuclei were labelled with DAPI and FACS sorted for subsequent sequencing using the Chromium system from 10X Genomics (10X Genomics, Pleasanton, CA, USA). Analysis of these sequences are detailed in supplementary methods (Additional file 1: online resource 1).

### Bioassay for *n*-agrin induced AChR clustering

Primary myogenic cells were seeded at a density of 1 × 10^5^ cells/well into 12 well tissue culture plates (Corning, AZ, USA) containing 13 mm diameter coverslips coated with 0.01% gelatin. When cells reached 80% confluence, growth medium was replaced with differentiation medium. Mature myotubes were treated with differentiation medium containing 10 nM *n*-agrin (R&D systems, USA, 50-AG-100) for 4 h at 37 °C and 5% CO_2_ [41]. Following treatment, cultures were rinsed with PBS then incubated with Alexa-488 α-bungarotoxin for 1 h to label surface AChR clusters [41]. Cultures were then washed with PBS and processed for Desmin immunostaining (detailed in supplementary methods, Additional file 1: online resource 1). *N*-Agrin assays were conducted over 3 coverslips (technical replicates) for each biological replicate. Images were acquired using a Leica DM2500 fluorescence microscope with commercial imaging software (LAS X, Leica Microsystems) using a 20 × objective. Three microscopic fields of myotube segments were imaged at random per coverslip. The exposure times for all images across all coverslips was kept constant. Antibody details are listed in Additional file 1: Table S4 (Additional file 1: online resource 2).

The area and number of AChR clusters were quantified with Fiji software (Fiji, RRID:SCR_002285; NIH, version 1.52e; [42]). The criteria for including a segment of myotube in AChR cluster counts were: expression of desmin, and ≥ 3 DAPI-stained nuclei per segment spanning over 80% of the 20 × visual field: 11,142 × 900 μm [41, 43]. Briefly, images were split into their respective channels and intensity thresholds were set to isolate the AChR clusters on myotubes from background artifacts. The *Analyse Particles* function was used to quantitate the areas and mean pixel intensities for the isolated clusters. Clusters smaller than 15 pixels (which might arise from digital camera noise) were excluded from analysis. Areas of AChR clusters measured in pixels were converted into µm^2^ and binned into multiples of 5 µm^2^ in a frequency histogram using GraphPad Prism (version 8). Investigators were kept blinded to the source of the cultured myotubes until all images were acquired and their respective analyses completed.

### Immunostaining of muscle biopsies

A portion of the collected muscle biopsies (non-MND and MND) were fixed in 4% PFA in PBS for 60 min at room temperature. Samples were then washed in 0.1 M Glycine in PBS and divided into two portions. One portion was processed for wholemount immunostaining, and the second portion was processed for cryosectioning. For wholemount immunostaining, teased muscle fibers were stained for postsynaptic AChRs with α-bungarotoxin and motor nerve terminal endings with a cocktail of rabbit anti-neurofilament 200 plus rabbit synaptophysin (see supplementary methods, Additional file 1: online resource 1). Image acquisition details and analyses of intact NMJs are also given in supplementary methods (Additional file 1: online resource 1).

For cryosectioning, muscle fragments were immersed in 15% sucrose in PBS overnight at 4 °C followed by 30% sucrose in PBS at 4 °C. They were then embedded in OCT compound (Fisher Scientific, USA), and frozen in liquid nitrogen super-chilled isopentane (Fisher Chemicals, USA). Muscle blocks were cryosectioned (L/S) at 20 µm and processed for immunostaining to locate MuSK using either sheep or rabbit anti-MuSK and AChRs with Alexa 555 α-bungarotoxin, at the motor endplate (see supplementary methods, Additional file 1: online resource 1). MuSK and AChR motor endplate staining areas were calculated by tracing the boundary of AChRs and MuSK immunoreactivity using the drawing tool in Fiji, followed by the measurement function to calculate the relative area in pixels. This method allowed us to assess the spread of MuSK staining beyond the AChR-rich region into the peri-synaptic area in MND muscle. Since images stained for MuSK-AChR were not taken with a fixed gain and black level it is not possible to directly compare the intensities of MuSK staining at the NMJs from MND versus non-MND muscle. We acknowledge that our area measurements are at best semi-quantitative, and thus we have not performed statistical analyses on these images. Antibody details are listed in Additional file 1: Table S4 (Additional file 1: online resource 2).

### Western blotting

Human myotubes were lysed in 50 mM Tris–HCL, 150 mM NaCl, 10 mM NaF, 10 mM Na_4_P_2_O_4_, 1 mM Na_3_VO_4,_ 1% NP40 and protease inhibitor (Roche, Basel, Switzerland). Protein concentrations were determined using bicinchoninic acid assays. Protein lysates were resolved by SDS-PAGE (8% or 12% acrylamide/bis acrylamide) and were transferred onto a nitrocellulose membrane by wet transfer. Membranes were blocked in 5% (w/v) skim milk in TBST (10 mM Tris–HCL, pH 7.5, 150 mM NaCl, and 0.1% Tween 20) and incubated with primary antibodies to MuSK, LRP-4, anti-Dok7 and Cav3 overnight at 4 °C. Membranes were then incubated with appropriate HRP-conjugated secondary antibodies (HRP anti-mouse [Sigma-Aldrich, Merck-Milllipore 12-348] or HRP anti-rabbit [Sigma-Aldrich, Merck-Millipore AP106P] in 1% (w/v) skim milk in TBST) for 1 h at room temperature. Enhanced chemiluminescence detection was conducted in a mix of Clarity and Clarity Max substrate solution from Bio-Rad (ratio 1:1). Chemiluminescence was imaged on a LI-COR Odyssey, and densitometrical values of bands were quantified arbitrarily using the Image Studio Software (LI-COR Biosciences, Lincoln Neb, USA). Anti-Tubulin was used as the loading control for the normalization of all lanes. Antibody details are listed in Additional file 1: Table S4 (Additional file 1: online resource 2).

### Statistics

All data were assessed for distribution using Shapiro–Wilk tests of normality. Data that passed this test were analyzed using one-way ANOVA with Bonferroni’s multiple comparisons test, two-way ANOVA followed by Bonferroni’s multiple comparisons test, or un-paired *t* test as appropriate. Skewed data were log transformed and assessed by two-way ANOVA followed by Bonferroni’s multiple comparisons test. GraphPad prism versions 8.0 and 9.0 were used for all analyses and the construction of graphs. Data are presented as mean ± SD. Differences were considered statistically significant when *p* ≤ 0.05 (where *p ≤ 0.05, **p ≤ 0.01, ***p ≤ 0.001, ****p ≤ 0.0001). Analysis of single nuclei RNA sequencing data was conducted using R (version 4.1.0), as detailed in supplementary methods (Additional file 1: online resource 1).

### Study approval

This study was approved by the Royal Brisbane and Women’s Hospital (RBWH) and the University of Queensland (UQ) Human Ethics committees (HREC/13/QRBWH/58, HREC/14/QRBWH/495 and NHMRC/UQ ethics 20119003063 and 2015000022), and was conducted in accordance with the Declaration of Helsinki Principles (Bulletin of the World Health Organization 2001) and the National Statement on Ethical Conduct in Human Research 2007 (Updated 2018; Australian National Health and Medical Research Council, the Australian Research Council and Universities Australia, Commonwealth of Australia, Canberra). All participants provided written informed consent prior to study participation.

## Results

### Demographics and clinical assessment

Research participants were recruited from the RBWH MND clinic. Muscle biopsies were collected from two cohorts. The demographic and neurological assessment details for cohorts 1 and 2 are summarized in Tables 1 and 2 respectively. The first cohort comprised 9 individuals who had an open muscle biopsy for diagnostic purposes (Table 1). Of these, 6 had a final diagnosis of sporadic MND; 5 presented with spinal cord onset disease and 1 with a bulbar onset disease. The remaining 3 samples were obtained from non-MND muscle donors (non-MND other disease controls) with neuromuscular symptoms requiring a muscle biopsy for pathological investigation (other non-MND disease controls; Table 1).

The second cohort comprised 23 donors who each provided a punch biopsy (Table 2). This group comprised 10 individuals with a diagnosis of MND and 13 age-matched healthy control individuals. Of the MND participants, 7 had sporadic MND, 1 carried a mutation in the SOD1 gene, and 2 had a repeat expansion in the C9orf72 gene; 9 presented with spinal cord onset and 1 with bulbar onset. Controls for cohort 2 were recruited from a convenience sample of family and friends of MND participants.

### MND muscle presents with an increased proportion of slow type fibers and fibers with low diameter

**Fig. 1:**
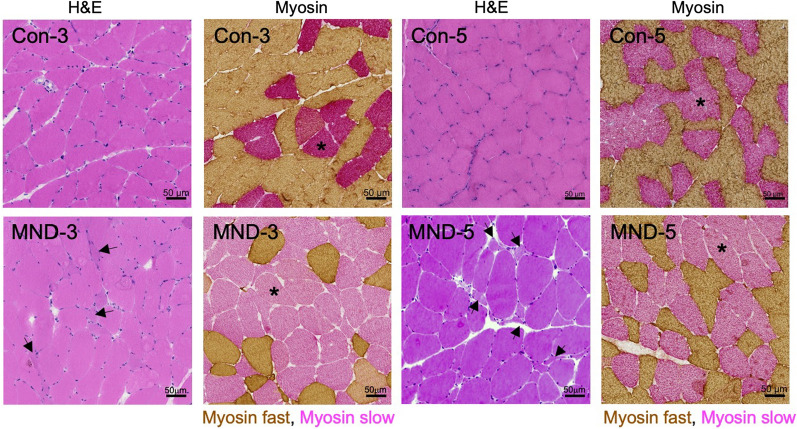
Muscle fiber type grouping occurs in MND patients. Sample cross sections of muscle from non-MND (Con-3 and -5) and from MND patients (MND-3 and -5). Sections were stained with H&E or combination anti-fast plus anti-slow myosin staining (brown stain and red stain respectively). Sections from MND-3 and -5 muscles reveal more grouping of slow myosin fibers (type I fibers;* *red stained fibers in 2nd and 4th panels of second row*) compared to those from Con-3 and -5, top row. Examples of atrophied (small) muscle fibers can also be seen in the MND sections (*arrows*). Scale bars = 50 μm

Pathological analyses of surgical muscle samples collected from 3 non-MND donors (Con-3, -4 and -5) revealed no overt muscle damage, with a pre-dominance of type II muscle fibers (Fig. 1*top row*; Additional file 1: Table-S1; and Additional file 1: Figure S1 (Additional file 1: online resource 2)). By contrast, increased proportions and grouping of slow type fibers was observed in all 6 surgical biopsies from individuals with MND, indicative of progressive denervation/reinnervation (Fig. 1*bottom row*; Additional file 1: Table S1; and Additional file 1: Figure S1 (Additional file 1: online resource 2)). These MND muscle samples also displayed fibers with small diameters, consistent with fiber atrophy (Fig. 1, arrows). Thus, our results reveal that MND muscle is characterized by an increased proportion of slow type fibers and those with smaller diameter.

### Neuromuscular synapses disassemble in MND muscle

**Fig. 2:**
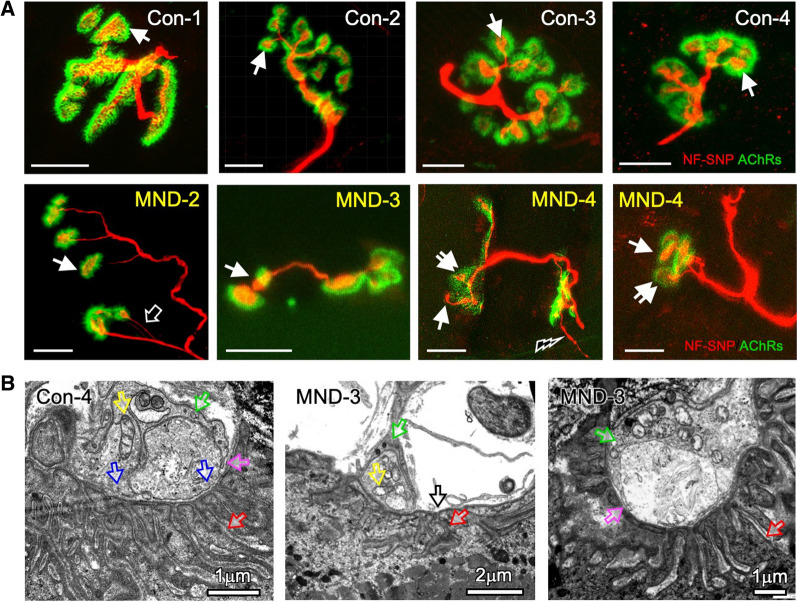
Muscle denervation-reinnervation and NMJ disassembly is evident in MND patients. **A** Maximum projection images of individual NMJs from non-MND and MND muscle biopsies. Motor endplates from four non-MND patients (Con-1, -2, -3 and -4) displayed presynaptic motor terminal endings (*red bulbous structures*), surrounded by dense clusters of postsynaptic AChRs (*green halos, white arrows*). NMJs from three MND patients (MND-2, -3 and -4) display varying stages of disassembly indicated by a reduced density or complete loss of postsynaptic AChR clusters that normally surround the nerve terminal endings (*single arrows*); shrinkage of nerve terminal relative to AChR cluster area (*double arrows*); terminal axonal thinning and sprouting (*black arrow and lightning bolt respectively*). NF-SNP = neurofilament and synaptophysin immuno-stain; AChR = 488 Alexa α-bungarotoxin stain. Scale bars = 10 µm. **B** Transmission electron micrograph of an NMJ from a non-MND subject (Con-4) displays the motor nerve terminal filled with synaptic vesicles (*blue arrows*), intact mitochondria (*yellow arrow*), and a terminal Schwann cell (*green and* *pink arrows in all panels*) demarcating the pre- and postsynaptic membrane apposition. Deep infoldings of the opposing postsynaptic muscle membrane (postsynaptic junctional folds) contain basal lamina (*red arrows in all panels*). NMJs from MND-3 (middle panel) shows motor terminal and a flattened motor endplate, with no overlying nerve terminal (*black arrow*), and signs of damaged mitochondria within the terminal ending (*yellow arrow*). Right panel reveals terminal Schwann cell invasion into the synaptic cleft (*green and arrows*)

We next examined neuromuscular synapses at the morphological level in non-MND and MND muscle biopsies; a total of 132 NMJs were examined from four non-MND and three MND donors (Tables 1 and 2). Examination of MND muscle samples, revealed disassembly of NMJs when compared to non-MND muscle (Fig. 2a). For non-MND muscle, 36 NMJs were examined across three *Vastus Lateralis* muscles, and 19 NMJs in one *Deltoid* muscle (total 55 non-MND NMJs). Non-MND muscle exhibited the expected *en grappe* (bunch of grape-like) NMJs [31, 44] with 92% being classified as normal (Table 3). They consisted of a single primary motor axon branch forming 2–3 secondary axonal branches. The bulbous (*bouton*) ending of each secondary branch was surrounded by a dense halo of acetylcholine receptors (AChRs; Fig. 2a *top row*; Additional file 1: Figure S2A–C (Additional file 1: online resource 2)). Approximately 8% of NMJs showed signs of reinnervation and/or partial denervation (Table 3). There was ~ 60% overlap of presynaptic nerve terminal and postsynaptic AChR labeling (Table 4 and Additional file 1: Table S2 (Additional file 1: online resource 2).

**Table 3:** Status of NMJs analyzed by confocal microscopy per donor biopsy

Donor ID and disease status	Fully Innervated (%) of total NMJs/Donor	Partially denervated-reinnervated (%) of total NMJs/Donor	Denervated (%) of total NMJs/Donor
Con-1	2/2 NMJs (100%)	0/2 NMJs	0/2
Con-2	12/12 NMJs (100%)	0/12 NMJs	0/12
Con-3	17/19 NMJs (89%)	2/19 NMJs (11%)	0/19
Con-4	20/22NMJs (90%)	2/22 NMJs (10%)	
Con-5	10/12 NMJs (83%)	2/12 NMJs (17%)	0/12
Con NMJ Mean %	92.4%	7.6%	0%
MND-2	4/13 NMJs (30%)	9/13 NMJs (70%)	0/13 NMJs
MND-3	5/40 (12%)	20/40 (50%)	15/40 NMJs (38%)
MND-4	0/24 (0%)	20/24 (83%)	4/24 (17%)
MND NMJ Mean %	14%	67%	18%

NMJ status is expressed as a fraction of classified NMJ over the total number of NMJs analyzed per donor muscle biopsy

**Table 4:** Morphometry of NMJs from confocal microscopic images

Morphological variable	non-MND *n* = 5 mean ± SD	MND *n* = 3 Mean ± SD	*P* value un-paired *t* test
Primary Motor axon thickness (μm)	1.546 ± 0.5	1.257 ± 0.7	n. s. *P* = 0.44
Motor axon terminal sprout thickness (μm)	0.926 ± 0.4	0.73 ± 0.4	n.s. *P* = 0.49
Motor Nerve terminal Bouton size (μm^2^)	8.03 ± 1.7	5.01 ± 0.8	* *P* = 0.034
Total NT area (μm^2^)	74.33 ± 10.3	36.71 ± 12.2	** *P* = 0.0033
AChR cluster patch size (μm^2^)	13.82 ± 2.2	18.82 ± 7.9	n.s. *P* = 0.36
Total AChR area (μm^2^)	126.5 ± 17.9	138.0 ± 26.9	n.s. *P* = 0.44
% of Motor nerve terminal to AChR overlap	60.14% ± 2.3	29.47% ± 7.1	*** *P* = 0.0001

^*^, **, *** Unpaired t – test; n.s. = not significant

Postsynaptic AChR clusters were used to identify a total of 77 motor endplates from MND samples. Of these, 58 displayed a motor nerve terminal whereas 19 endplates revealed no overlying motor nerve terminal (Table 3). Evidence of partial denervation and/or reinnervation was observed in 67% of NMJs. A further 18% were classified as denervated. On average, nerve terminal staining covered ~ 30% of the postsynaptic AChRs. This was due, in part, to a significant reduction in the average size of the nerve terminal boutons (Table 4 and Additional file 1: Table S2 (Additional file 1: online resource 2)). Labeling for AChRs appeared more diffuse, which could reflect either a flattening of the postsynaptic membrane, or a loss of AChRs from postsynaptic regions (*arrows in* Fig. 2a *lower row*; Additional file 1: Figure S3A–C (Additional file 1: online resource 2)). We also observed terminal sprouting and some axonal thinning (Fig. 2a *lower row*; Additional file 1: Figure S3A–C (Additional file 1: online resource 2)), consistent with previous studies [4, 27], but across all NMJs the average difference in axonal diameter was not statistically significant (Table 4 and Additional file 1: Table S2 (Additional file 1: online resource 2)). There was evidence of separation of individual *en grappe* terminal endings (Fig. 2a, *see* MND-2*;* and Additional file 1: Figure S3A–C (Additional file 1: online resource 2)), but this did not appear to be a distinguishing feature of NMJs from MND patients.

Transmission electron microscopy revealed examples of ultrastructural abnormalities consistent with impairments at NMJs from MND patients [4]. These include flattening of the postsynaptic membrane not occupied by a motor nerve terminal (*black arrow in* Fig. 2b *middle panel*), pre-synaptic mitochondria containing clear vacuoles, and partial invasion of terminal Schwann cell processes into the synaptic cleft (Fig. 2b, *yellow and pink arrows respectively in middle and right panels compared to a non-MND NMJ left panel*). However, the postjunctional folds and synaptic basal lamina appeared to be structurally similar across NMJs from non-MND and MND muscle (*red arrows in* Fig. 2b) [45]. Taken together, our data indicate that there are significant alterations in NMJ structure in MND.

### MuSK localization is altered at the motor endplate of NMJs in MND muscle

**Fig. 3:**
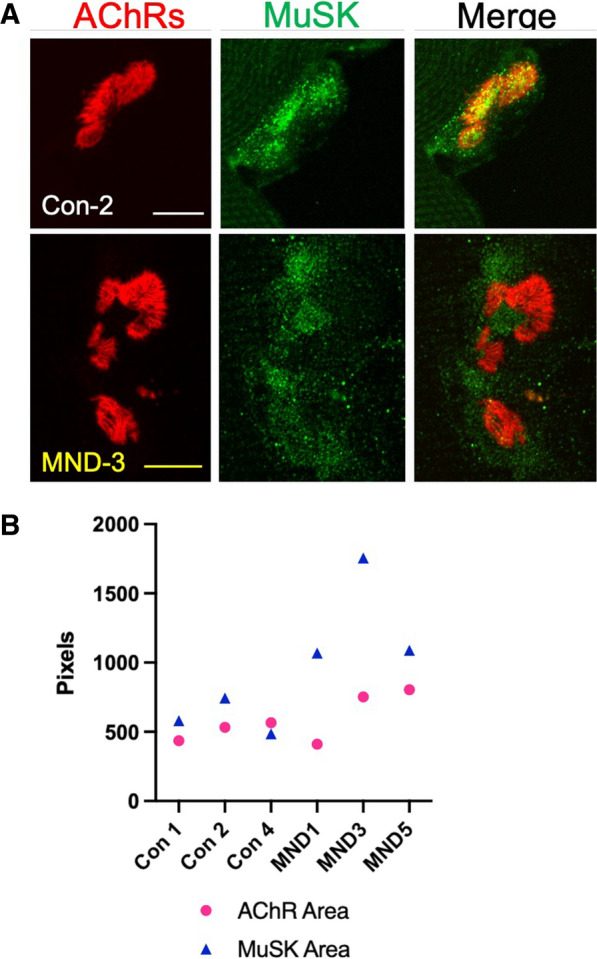
MuSK staining is more diffuse relative to the AChR-rich motor endplate. **A** Compares the staining areas for acetylcholine receptors (AChRs) and MuSK at the motor endplate from a non-MND (Con-2) donor, and from an MND patient (MND-3). At the motor endplate of MND-3, MuSK staining occupied a larger area compared to the AChR area. By contrast, the MuSK area was a closer match to AChR at the Con-2 motor endplate. **B** Shows the relative pixel area occupied by AChR and MuSK staining per motor endplate from non-MND donors (Con-1, -2, and 4) and MND patients (MND-1, -3 and -5). At non-MND motor endplates, there appeared to be tight co-localization of AChR and MuSK staining as depicted by the closeness of their respective AChR and MuSK data points. By contrast at MND motor endplates, MuSK occupied a larger area of staining compared to AChR area. Scale bar = 20 μm

Given the central role of MuSK signaling in stabilizing NMJs, we compared the expression and localization of postsynaptic MuSK at motor endplates in open muscle biopsy specimens from MND and non-MND individuals. At motor endplates within MND *vastus lateralis* muscle, MuSK immunostaining occupied a larger area compared to its respective AChR stained motor endplate area. By contrast motor endplates within non-MND *vastus lateralis* muscle, showed MuSK immuno-staining to be more restricted to the AChR labelled motor endplate (Fig. 3, and Additional file 1: Figure S4 (Additional file 1: online resource 2)). These observations suggest that MND has somehow induced the delocalization of MuSK from the motor endplate (postsynaptic) region.

### Impaired nerve induced AChR clustering in muscle cells grown from MND satellite cells

**Fig. 4:**
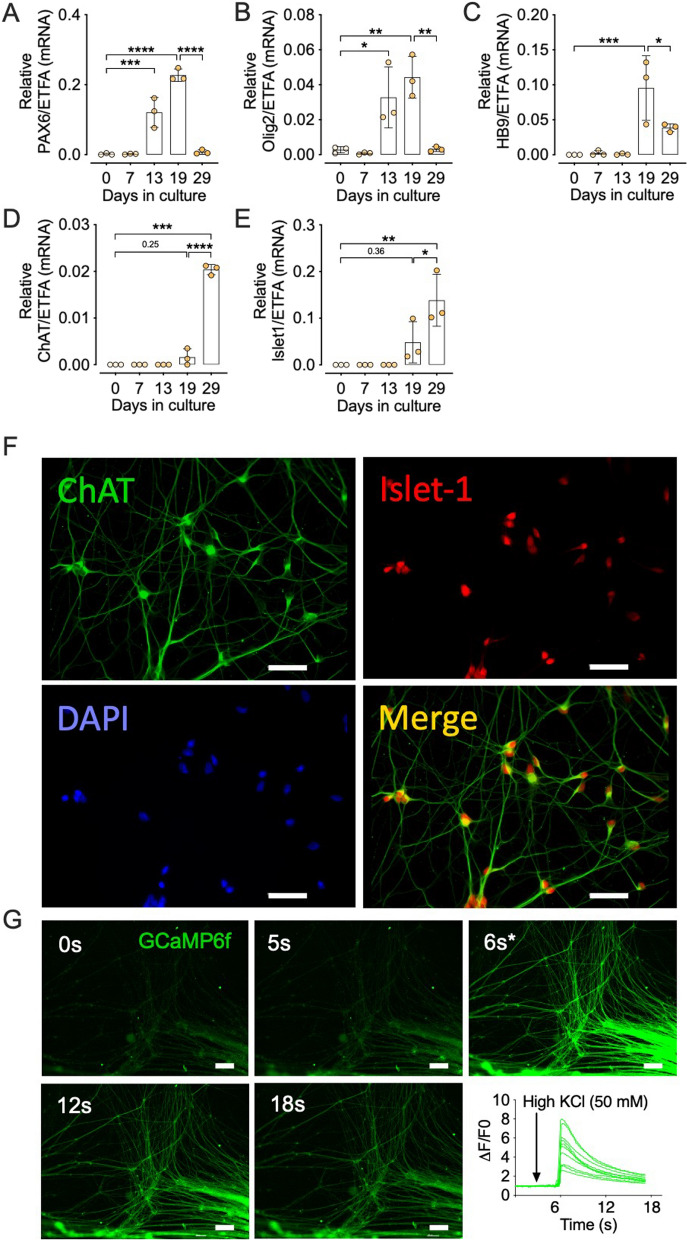
Differentiation of H9 human embryonic stem cells in culture. **A–E** Quantification of mRNAs encoding Pax6 (**A**), Olig2 (**B**), HB9 (**C**), ChAT (**D**) and Islet-1 (**E**) (*n* = 3). **F** Sample visual field of differentiated H9 cells labeled for ChAT, Islet-1, and nuclei (DAPI). Lower right panel shows the merge of ChAT and Islet-1 fluorescence. Scale bar for F = 100 μm. **G** A time series showing the calcium flux in differentiated neurons after addition of 50 mM KCl at time zero. Scale bar for **G** = 160 μm. The inset graph shows the time course of intracellular Ca^2+^ mobilization revealed by Ca^2+^ sensor fluorescence GCaMP6f. ΔF/F0 represents the fluorescence intensity relative to the initial fluorescence intensity. Data presented in **A–E** are means ± SDs, expression data analyzed using one-way ANOVA with Bonferroni’s multiple comparisons test. *p ≤ 0.05, **p ≤ 0.01, ***p ≤ 0.001, ****p ≤ 0.0001

We developed a human motoneuron-muscle co-culture system to investigate the interactions between healthy motor nerves and muscle cells cultured from MND donors. Human H9 embryonic stem cells (hESCs) [37] were stably transfected with a GcaMP6f calcium reporter [46] and differentiated into motor neurons [38]. Over a period of 29 days in culture, cells displayed an ordered temporal expression of molecular markers defining commitment to spinal cord motor neurons [47–49]. The expression of paired box transcription factor Pax6, and the basic helix loop helix transcription factor Olig 2 were detected at day (D) 13, peaking at D19 and declining by D29 (Fig. 4a, b). Peak expression of Pax6 and Olig2 coincided with transient upregulation of the early motor neuron transcription factor HB9 at D19 (Fig. 4c). By D29, expression of HB9 had dropped significantly, reminiscent of the peak of HB9 expression in mammalian spinal cord motor neurons just prior to birth, and its subsequent decline post birth [47, 50–53]. Expression for choline acetyltransferase (ChAT; Fig. 4d) and Islet-1 (Fig. 4e) were low at D19, but were significantly upregulated by D29, indicating molecular maturation of motor neurons [47, 54, 55]. Expression of ChAT and Islet-1 protein was confirmed by immunofluorescent staining. By D29, ChAT and Islet-1 immunolabeling were observed in all neurons, with ChAT being expressed throughout the cytoplasm and Islet-1 being expressed in the nuclei (Fig. 4f). To assess the response of these hESC-derived motor neurons to depolarization, cultures were subjected to a brief pulse of KCl (50 mM). Approximately 6 s later, we observed a rapid and transient rise in levels of intracellular calcium (GcaMP6f fluorescence) which returned to baseline over the course of 12 s (Fig. 4g). Together these observations demonstrate the successful differentiation of H9 hESCs into functional human motor neurons expressing postnatal motor neuron markers.

**Fig. 5:**
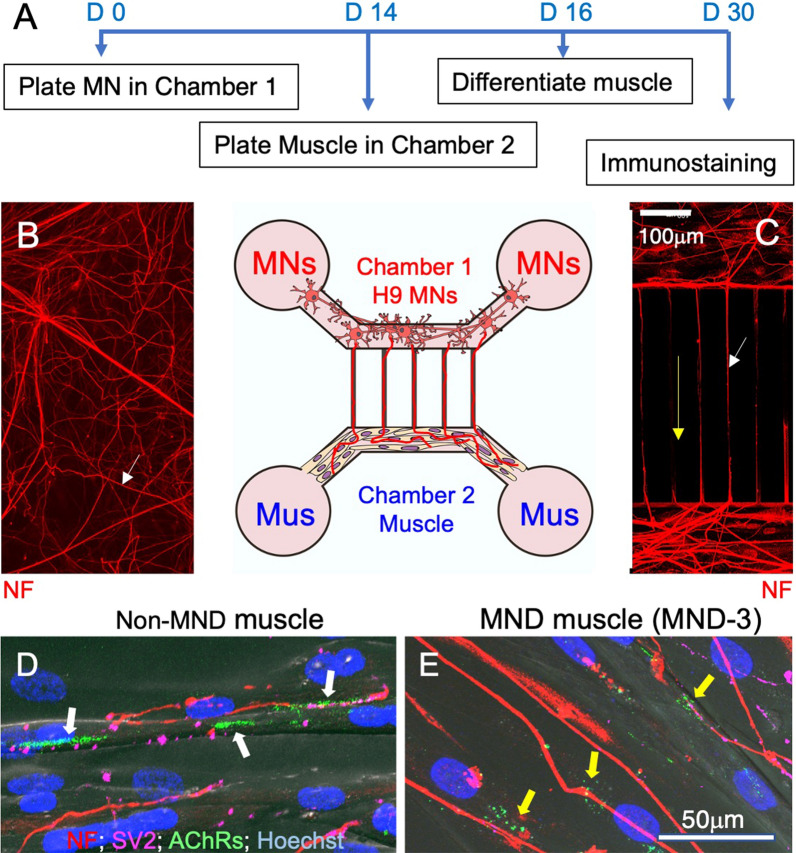
Muscle grown from MND patients responded poorly to human motor axons. **A** Timeline for human derived MNs innervating human skeletal muscle in a microfluidic culture system. **B** Representative visual fields showing axons of H9 stem cell-derived motor neurons (H9 MNs) immunostained for neurofilament (NF, *white arrow*). **C **MN axons grew along the microgrooves (*white arrow*) to enter the muscle chamber. Direction of axonal growth is indicated by the yellow arrow. **D** Representative visual field from the muscle chamber, showing neurofilament (*red*) and SV2 (*pink*) positive H9 motor axons intermingled with muscle cells grown from a non-MND donor. White arrows indicate large AChR clusters (*green*) that were located close to NF/SV2-positive axons. ** E** Muscle cells sourced from an MND patient displaying small AChR clusters (*green, yellow arrows*) adjacent to NF/SV2 positive axons. Muscle cell nuclei in **C**, **D** were labelled with Hoechst 33,342 (*blue*)

Motor neuron-muscle co-cultures were established to assess whether H9 human motor neurons could induce the clustering of AChRs in human muscle cultures (Fig. 5). H9 hESCs were seeded into chamber 1 of a microfluidic device and differentiated into motor neurons over the ensuing 14 days (D0 to D14), with axons extending 450 µm towards the second chamber of the microfluidic device (Fig. 5a, *arrows in* b and c). At D14, muscle satellite cells derived from muscle biopsies were seeded into the second chamber of the microfluidic device and subsequently differentiated into multi-nucleated muscle fibers (myotubes). Over the next 16 days (D14 to D30), axons intermingled with muscle cells sourced from non-MND or MND muscle (Fig. 5d, e, respectively). Muscle cells from non-MND donors revealed large clusters of AChRs near axons (labeled with a neurofilament (NF)/synaptic vesicle 2 (SV2) antibody cocktail; (Fig. 5d, *white arrows*). By contrast, muscle cells derived from MND muscle exhibited no large AChR clusters near NF/SV2 positive axons, with only small clusters of AChRs being observed (Fig. 5e, *yellow arrows*).

**Fig. 6:**
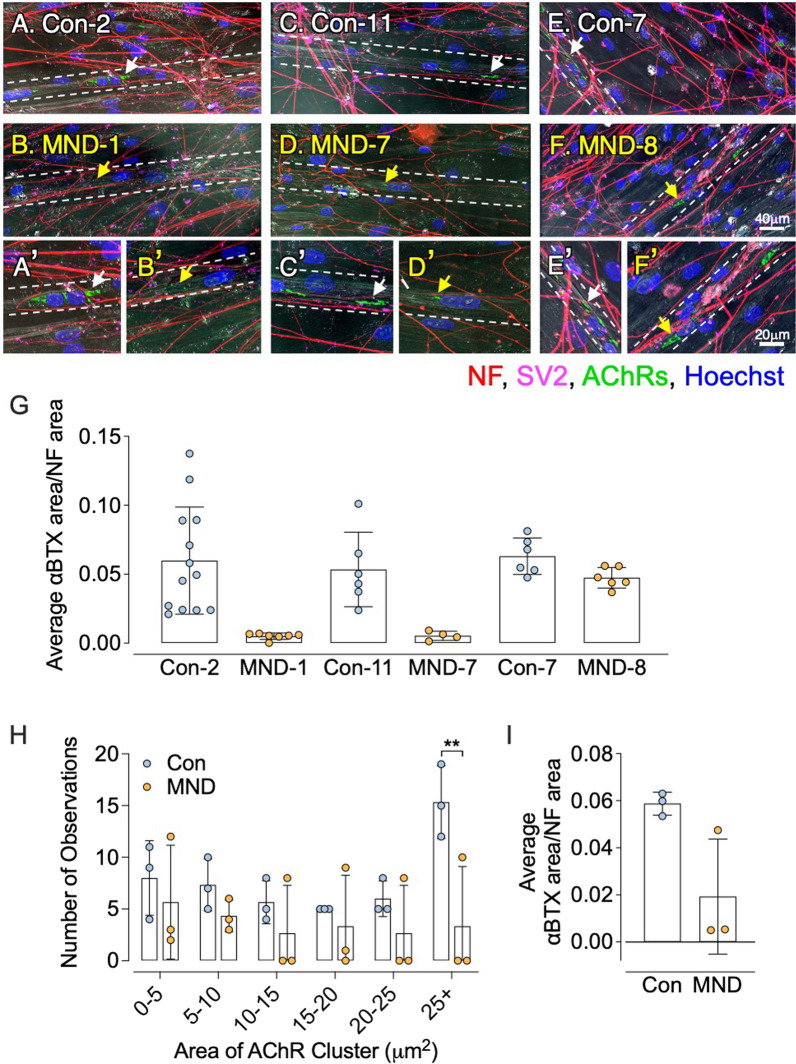
Nerve-induced acetylcholine receptor clustering is impaired in muscle from MND patients. **A–F** Sample visual fields showing human myotube cultures innervated by H9 human axons labeled for neurofilament-SV2 (*red-pink*) and AChRs clusters (*green*). Three paired sets of non-MND versus MND muscle cells (matched for sex and approximate age) are compared (A *vs* B, C *vs* D, E *vs* F). Insets (**A′, B′, C′, D′, E** and **F′**) show the AChR clusters indicated by arrows at higher magnification. Dotted white lines indicate the edge of the myotubes. White arrows in **A′, C′** and **E′** indicate large AChR clusters proximal to NF-SV2 labeled motor axons. Yellow arrows in **B′**, and **D′** indicate small AChR clusters close to NF-SV2 labeled motor axons. Yellow arrows in **F′** show large AChR clusters close to NF-SV2 labeled motor axons. Scale bars for **A**–**F** = 40 µm, and for **A′**–**F′** = 20 µm. **G** Quantitation of the area of AChR clusters, expressed as a fraction of the total area of axons averaged from 3 visual fields per MN-muscle sample, as per [56]. **H** Frequency histogram showing a significantly greater number of large AChR clusters (> 25 µm^2^) on myotubes of control cultures compared to MND cultures (*n* = 3 patients). **I** Total area of AChR clustering (large plus small clusters) per microscope field. Data points show results from individual data presented in **G**, (*n* = 3 patients). Data presented as mean ± SD. For **H**, data was analyzed using two-way ANOVA, followed by Bonferroni’s multiple comparisons test (**p ≤ 0.01)

In quantifying the apparent poor AChR clustering response of MND muscle cells when compared to non-MND muscle, we sought to control for possible confounding variables, such as age and sex, that might affect the myogenic capacity of muscle. Three paired sets of non-MND and MND muscle cells (matched for sex and approximate age) were co-cultured as described above, and the area of AChR clusters on myotubes was quantified relative to the area contacted by NF/SV2 positive axons (as per [56]). For two of the three MND/non-MND sample pairs the ratio of AChR cluster area to NF/SV2 axon area was significantly higher in the non-MND sample (Fig. 6a–g). Myotubes generated from MND-1 and MND-7 displayed very few large AChR clusters when compared to myotubes generated from age- and sex-matched controls (Con-2 and Con-11 respectively, Fig. 6a–d, Fig. 6g). In the case of myotubes generated from MND-8, there was no significant difference in AChR cluster area when compared to myotubes generated from an age- and sex-match control (Con-7, Fig. 6e–g).

We then pooled this AChR cluster size data to generate frequency distributions. The number and size of AChR clusters were only assessed on myotubes that spanned the visual field. The average size of AChR clusters was lower in MND myotubes when compared to non-MND myotubes. MND myotubes were deficient in the largest size category of AChR clusters (those > 25 µm^2^; Fig. 6h, n = 3, ** *P* < 0.0001, two-way ANOVA). The average ratio of total AChR cluster area to total NF/SV2 axon area appeared lower for MND myotubes compared to non-MND myotubes (Fig. 6i). In summary, myotubes cultured from MND patients appeared to produce fewer large AChR clusters in response to innervation by human motor neurons.

### Muscle stem cells isolated from MND donors mature slower than those from non-MND donors

**Fig. 7:**
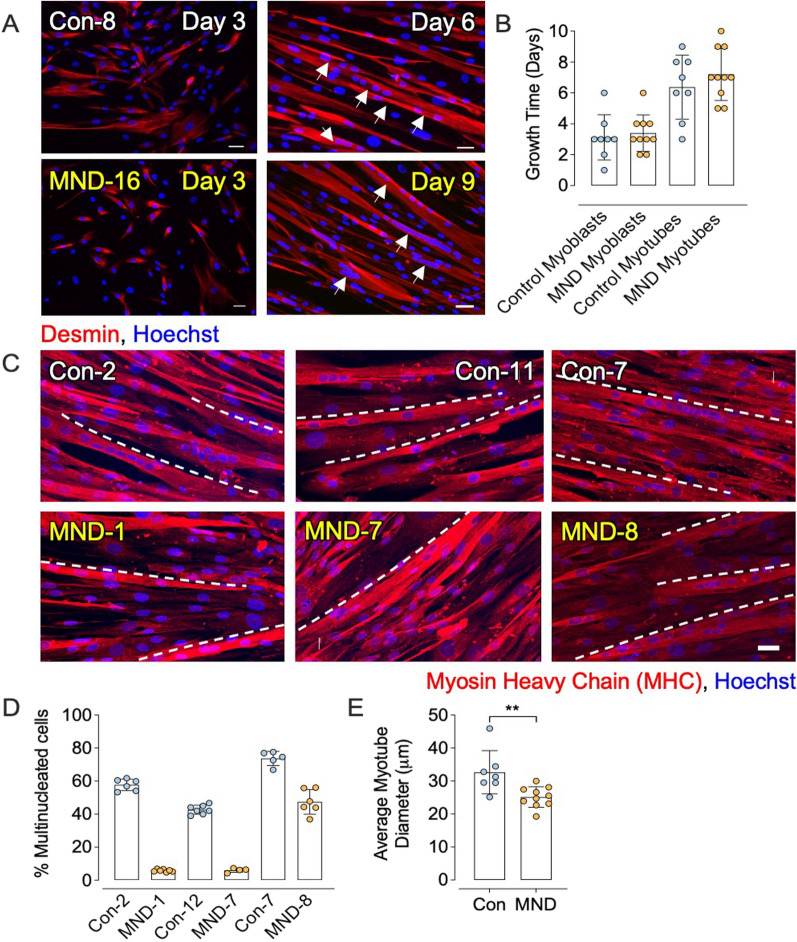
Muscle cells from MND patients show similar proliferation and fusion capacity, but a lower plate density. (**A** *left panels*) Desmin staining (*red fluorescence*) identifies mono-nucleated muscle cells sourced from a non-MND donor (Con-8) and a MND donor (MND-16) at 3 days in culture (~ 80% confluency). (**A** *right panels*) Desmin-labeled multinucleated myotubes from Con-8 and MND-16 (*arrows*). Scale bar = 20 µm. **B** First two bars show the number of days required for mononucleated cells to proliferate and reach 80% confluency. Non-MND and MND sourced cells appeared to proliferate at a similar rate. The third and fourth bars compare the days required for myoblasts from non-MND and MND donors to fuse to form multinucleated myotubes. There was no difference between MND and non-MND in the time required for myotube formation (*n* = 8 for control and *n* = 10 for MND). **C** Shows representative visual fields of myosin heavy chain (MHC) positive myotubes from matched control (Con) donors and MND patients (Con-2/MND-1, Con-11/MND-7, Con-7/MND-8). White dashed lines delineate the upper edge of a myotube. Scale bar = 20 μm. **D** Percentage of MHC positive multi-nucleated myotubes (≥ 3 nuclei/visual field) that were averaged across 6 visual fields per muscle sample. All non-MND (control) muscle cultures produced more MHC positive myotubes per visual field, compared to cultures from MND patients. **E** Quantitation of myotube diameters. MND myotubes were slender compared to non-MND myotubes. Each symbol represents the average for a single non-MND or MND donor. All data presented as mean ± SD. **B** Data analyzed by two-way ANOVA followed by Bonferroni multiple comparisons test. **E** Data analyzed by unpaired *t* test, where **p ≤ 0.01

**Fig. 8:**
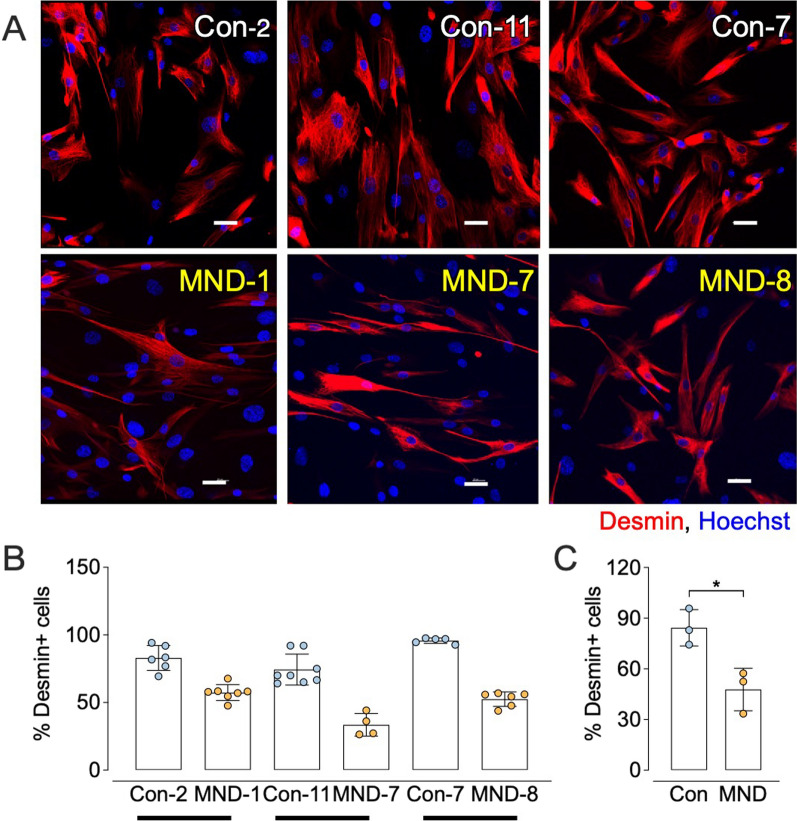
Cells cultured from muscle of MND patients produce fewer desmin-positive myoblasts, compared those from matched non-MND donors. **A** Representative visual field of desmin-positive cells cultured from matched control donors (Con, non-MND) and MND patients, black bars in B indicate the relative pairing of Con (non-MND) with MND samples. Scale bar = 40 μm. **B** Percentage of cells that stained positive for desmin, 3 days after removal of the growth medium (mean ± SD). Each symbol represents results from one non-MND or MND donor (averaged across 4–7 visual  fields per cultured muscle sample). Cells cultured from MND donor muscle revealed a lower percentage of myoblasts, when compared to their matched non-MND control muscle culture. **C** Pooled data showing a significantly lower percentage of desmin-positive cells in cultures from MND muscle compared to cultures from control (Con) muscle. All data presented as mean ± SD. For **C**, data analyzed by unpaired* t* test where *p ≤ 0.05

Since the maturity of myotubes can affect the expression of AChRs, MuSK and rapsyn, we examined the time-course of differentiation of muscle cells. The proliferation capacity of myoblasts was similar between non-MND and MND cultures. Myoblasts took ~ 3 days to reach 80% confluency (Fig. 7a *left panels*, b *left bars*) [57]. A further ~ 3 days was required for fusion of MND and non-MND myoblasts into multi-nucleated desmin-positive myotubes (Fig. 7a *right panels*, b *right bars*). However, there were fewer multinucleated myotubes per visual field compared to age- and sex-matched non-MND cultures (Fig. 7c, d). Moreover, the diameters of MND myotubes were narrower when compared to non-MND myotubes (Fig. 7e). The lower number (density) of myotubes generated in MND cultures could be due to the lower proportion of desmin-positive myoblasts compared to total cell numbers (Fig. 8).

**Fig. 9:**
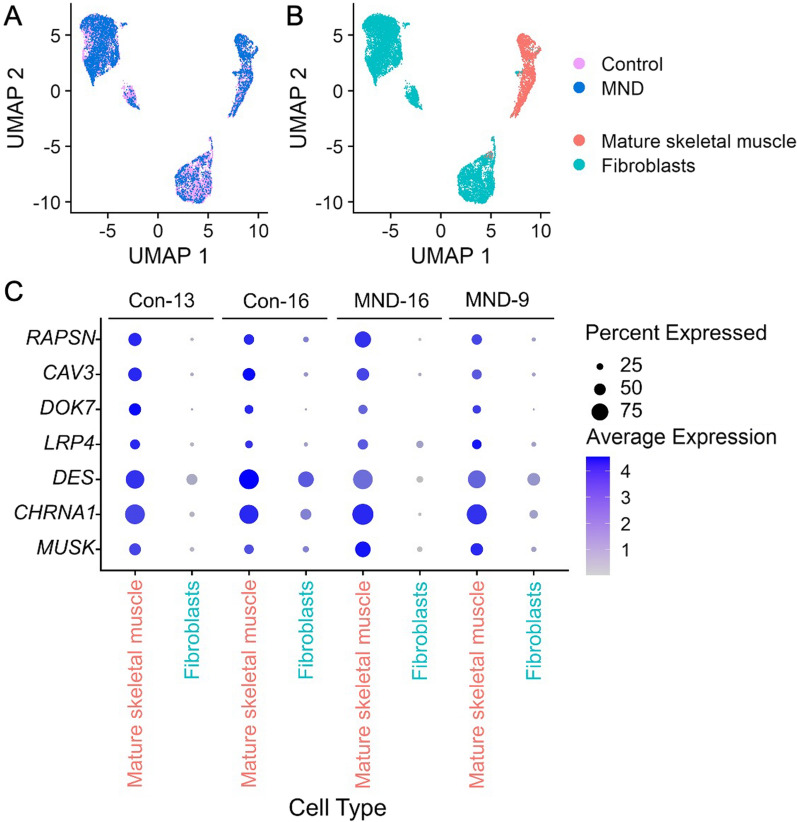
Transcriptomic analysis of muscle cell culture nuclei derived from human muscle stem cells identifies fibroblastic and muscle cell types with no apparent bias across MND status. **A** Whole-transcriptome expression profile patterns for individual nuclei derived from control (Con-13, Con-16) and MND donor (MND-16, MND-9) lines. Distinct expression profile patterns have been reduced to two-dimensions in this Uniform Manifold Approximation and Projection (UMAP) plot. Each point shows results for a single nucleus. MND and non-MND nuclei (pink and blue) are co-clustered in the UMAP, indicating similar patterns of whole-transcriptome expression between MND and controls. **B** Nuclei annotated for cell-type, identifying two distinct populations: mature skeletal muscle and fibroblasts. **C** Transcript dot plot comparing levels of expression for some key components of the *n*-agrin-MuSK signaling cascade in two MND samples versus two non-MND samples (see Additional file 1: Figure S5 for full data set). All four samples show transcripts consistent with their expression in muscle cells. Expression patterns of *n*-agrin-MuSK signaling transcripts appeared comparable between MND and control. Further, all samples do have nuclei that express transcripts associated with maturing muscle skeletal muscle

Given the lower proportion of myotubes in MND muscle cultures, we sought to characterize gene expression patterns in muscle cultures. We conducted single nuclei RNA-sequencing on differentiated cultures generated from two control donors and two MND donors. Cultures from each individual revealed shared cell types and similar whole-transcriptome gene expression profiles between control and MND muscle cultures, as illustrated by the mixing of nuclei in the Uniform Manifold Approximation and Projection (UMAP) plot (Fig. 9a). The heterogeneous clustering of nuclei is indicative of multiple cell types. We therefore used a single cell transcriptomic atlas of human skeletal muscle [58] as a reference to annotate the cell types of the single nuclei collected from control and MND muscle cultures [59]. Our analyses revealed two cell types in our cultures, fibroblast and myotubes (Fig. 9b). Consistent with this, analysis revealed expression of classical myogenic genes including dystrophin *(DMD)*, myocyte enhancer factor 2 (*MEF2A*)*,* titin *(TTN)*, myosin heavy chain (*MYH3*), desmin (*DES*), ryanodine receptor (*RYR1*) and myogenin *(MYOG)*—all similarly upregulated in nuclei identified as mature skeletal muscle (multinucleated myotubes). Overall, this indicates no overt differences in myogenic potential across MND and non-MND cultured muscle cells (Additional file 1: Figure S5 (Additional file 1: online resource 2)). Across myotube nuclei of both MND and non-MND lines, transcripts of the *n*-agrin-MuSK signaling pathway (e.g. *LRP4*, *MuSK, Dok7,* caveolin 3 *(CAV3)* and acetylcholine receptor alpha subunit (*CHRNA1*)) were found to closely mirror that of the above myogenic genes, with no obvious difference in their transcript expression across MND and control samples (Fig. 9c).

### Muscle cells cultured from MND donors respond poorly to *n*-agrin

**Fig. 10:**
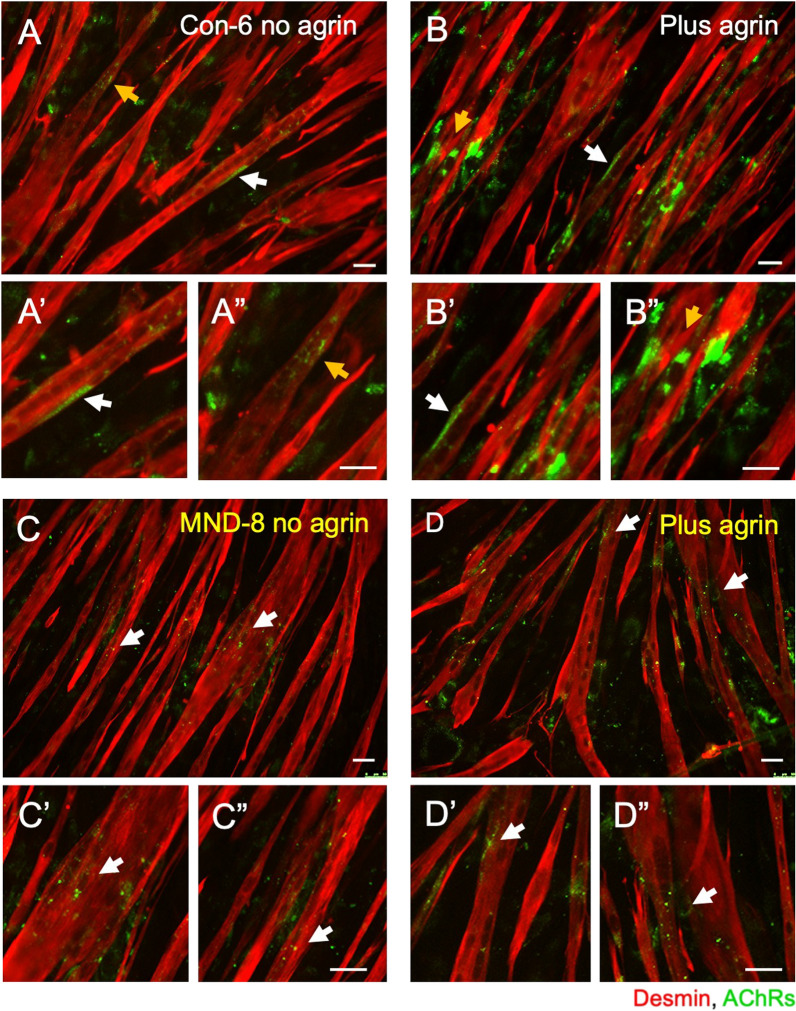
Sample images showing AChR clusters on myotubes cultured from non-MND and MND muscle before and after *n*-agrin treatment. **A** Sample field of desmin-positive myotubes cultured from muscle of non-MND patient Con-6, prior to *n*-agrin treatment. Small and occasional large AChR clusters are evident (*yellow* and *white* arrows respectively). **A′** and **A″** These AChR clusters at higher magnification. **B** Sample field of desmin-positive myotubes from Con-6 after *n*-agrin treatment. Large and small AChR clusters appear more numerous after *n*-agrin treatment (arrows and magnified views are shown in **B′** and **B″**). **C**, **D** Comparable sample fields of myotubes cultured from MND-8 (**C**) before and (**D**) after *n-*agrin treatment. Only small AChR clusters are visible both before and after n-agrin treatment (*white* arrows in C and D and their high-powered views: **C′**–**C″** no *n*-agrin, and **D′**–**D″** plus *n*-agrin). Scale bars = 50 µm

To test the possibility that *n*-agrin-MuSK signaling is impaired in MND muscle, we used a well- established muscle bioassay to induce AChR clusters with *n*-agrin. *N*-agrin was applied only when most myotubes in the culture reached a diameter of ~ 25–30 μm and spanned > 80% of the microscope field. Analysis was restricted to multinucleated, desmin-positive myotubes that spanned the visual field (~ 300 – 350 μm). Before treatment with *n*-agrin, myotubes generated from non-MND control donor muscle displayed small AChR clusters plus a few large AChR clusters (Fig. 10a). Treatment with *n*-agrin led to an increase in the number of large AChR clusters (Fig. 10b). By contrast, myotubes generated from MND donor muscle failed to respond to *n*-agrin. They displayed only small AChR clusters either with or without *n*-agrin treatment (Fig. 10c, d).

**Fig. 11:**
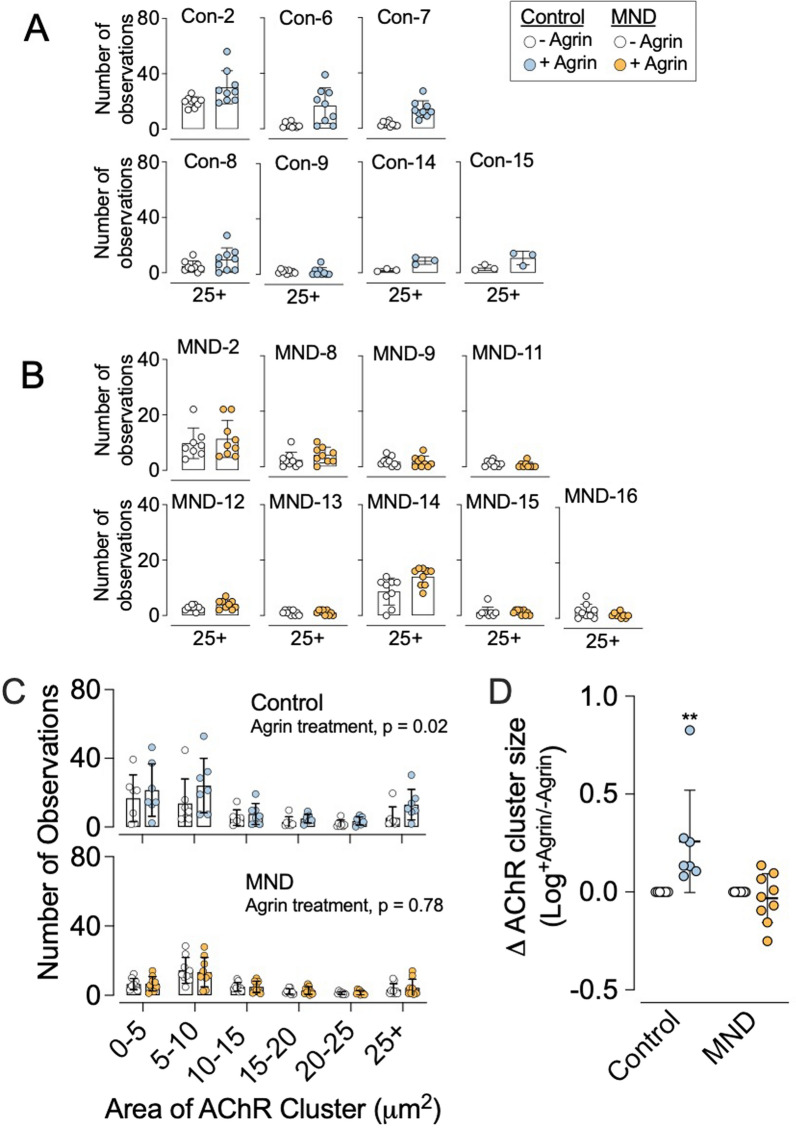
Muscle cells cultured from MND patients fail to grow AChR clusters in response to *n*-agrin treatment. **A** Myotubes cultured from individual non-MND donors formed large AChR clusters in response to *n*-agrin. Histograms compare the frequency of large (> 25 µm^2^) AChR clusters in cultures treated with (+) and without (−) *n*-agrin. Six out of seven non-MND donors showed an increase in the frequency of large AChR clusters in response to *n*-agrin treatment (exception being Con-9, see also Additional file 1: Figure S6). **B** Myotubes cultured from MND patients responded poorly to *n*-agrin. Apart from MND-14, none of the MND patient cultures showed any increase in the frequency of large (> 25 µm^2^) AChR clusters in response to *n*-agrin treatment. **C** Frequency distributions compare the sizes of AChR clusters on myotubes with and without *n*-agrin treatment. Each symbol represents the means ± SD for technical replicates; Additional file 1: Figure S6. (**C** *upper graph*) Myotubes cultured from non-MND (control) muscle with and without *n*-agrin treatment (pooled data from 7 donors). (**C** *lower graph*) Myotubes cultured from MND patient muscle with and without *n*-agrin treatment (pooled data from 9 patients: Additional file 1: Figure S7). **D** After n-agrin treatment, total area of clustered AChRs was significantly increased in control myotubes (*n* = 7, *blue and white symbols*). No significant change in total area of clustered AChRs was found in MND myotubes after *n*-agrin treatment (*n* = 9, *yellow and white symbols*; data presented as a log of plus (+) *n*-agrin over no (minus, −) *n*-agrin treatment). All data presented as mean ± SD and analyzed by two-way ANOVA with Bonferroni’s multiple comparison test where **p ≤ 0.01

Untreated cultures from seven different non-MND donors varied in the number of large (> 25 µm^2^) AChR clusters but, with one exception (Con-9), *n*-agrin treatment caused an increase in the number of large clusters in every control culture (Fig. 11a and Additional file 1: Figure S6 (Additional file 1: online resource 2)). Frequency distributions of pooled data showed that muscle cells grown from non-MND donors responded robustly to *n*-agrin treatment (*p* = 0.02, 2-way ANOVA, effect of *n*-agrin treatment) by increasing AChR cluster size (*p* < 0.001; 2-way ANOVA, effect of cluster size; Fig. 11c *upper graph*). Treatment with *n*-agrin increased the total AChR cluster area per visual field across all non-MND myotube samples (** *p* < 0.01, *n* = 7, 2-way ANOVA with Bonferroni’s multiple comparison, *compare blue data points vs white data points for control;* Fig. 11d). These observations are consistent with findings from a range of species in which *n*-agrin has been shown to cause a growth in AChRs clusters in cultured muscle [8, 9, 60–62].

By contrast, myotubes derived from MND donors showed no significant increase in the number of large AChR clusters (> 25 µm^2^) after treatment with 10 nM *n*-agrin (Figs. 11b and Additional file 1: Figure S7 (Additional file 1: online resource 2)). Frequency distributions of pooled data showed that muscle cells grown from MND patients did not respond to *n*-agrin treatment (*i.e.* no-effect of *n*-agrin treatment, *p* = 0.78, 2-way ANOVA, Fig. 11c *lower graph*). MND muscle cultures treated with *n*-agrin did not exhibit a significant increase in the total AChR area when compared to non-treated MND muscle cultures (*p* > 0.99, *n* = 10, 2-way ANOVA with Bonferroni’s multiple comparison, *MND orange data points vs white data points;* Fig. 11d). The failure of MND muscle cultures to increase AChR clustering in response to *n*-agrin suggests possible defects in the *n*-agrin-LRP4-MuSK signaling pathway that normally drives AChR cluster growth.

### MND muscle presents with altered levels of MuSK and associated proteins

**Fig. 12:**
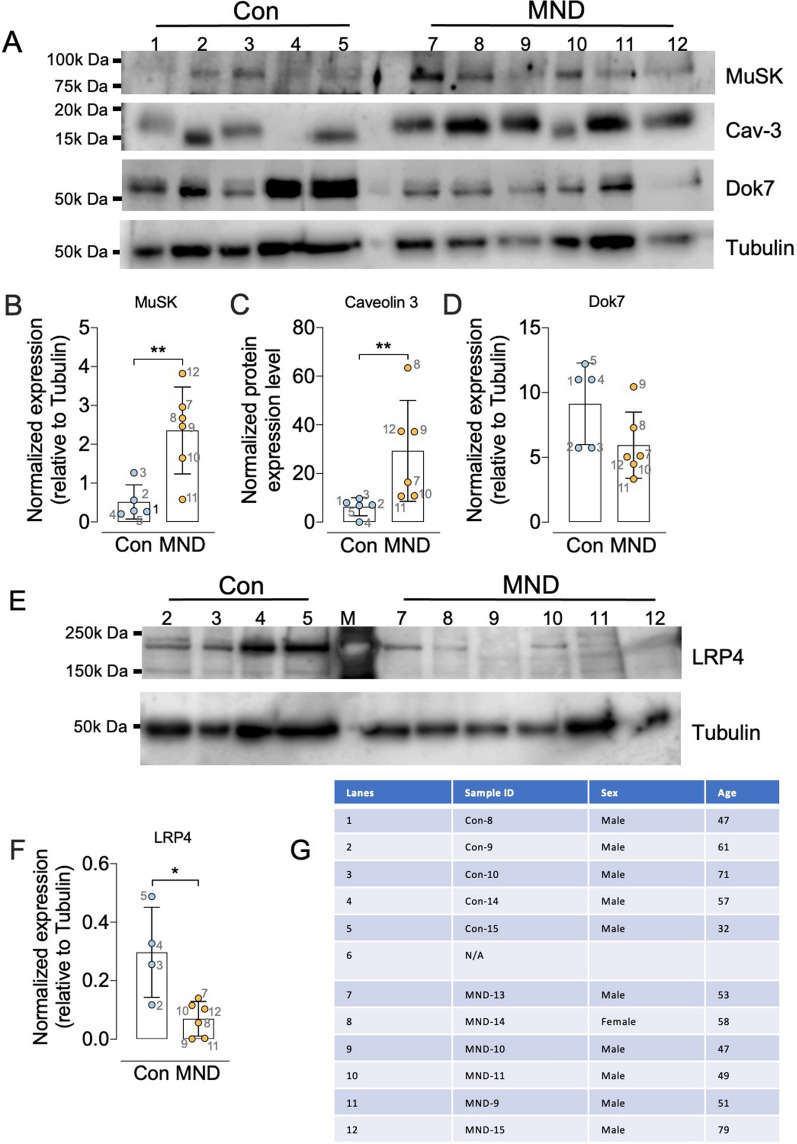
Levels of *n-*agrin effector proteins are altered in muscle cultures from MND patients. **A** Sample immunoblot comparing the relative intensity of MuSK, Caveolin-3 and Dok7 bands in myotube cultures derived from 4–5 non-MND donors and 6 MND patients. Antibodies revealed bands of the expected molecular weight for MuSK (~ 97 kDa), Caveolin-3 (~ 20 kDa) and Dok7 (~ 55 kDa). Each band was normalized to a tubulin loading control band from the same lane (shown beneath). Comparison of the normalized band intensities for **B** MuSK, **C** Caveolin-3, and **D** Dok7. **E** Sample immunoblot for LRP4 (~ 212 kDa). **F** Compares the normalized band intensities for LRP4 in non-MND and MND myotube cultures. Symbols each represent cultures from an individual donor (blue and yellow data). **G** Key showing the source of the samples run in the indicated lanes of the immunoblots. All data presented as mean ± SD and were analyzed using unpaired t-tests where *p ≤ 0.05 and **p ≤ 0.01

We next compared the levels of key proteins of the *n*-agrin-LRP4-MuSK pathway that drive AChR cluster stabilization. We observed higher levels of MuSK and Caveolin-3 in MND muscle cultures when compared to non-MND cultures (Fig. 12a–c, n = 5–6, **p = 0.008 and *p* = 0.04 respectively, unpaired two-tailed *t*-test). By contrast, LRP4 was significantly lower in MND muscle cultures when compared to non-MND cultures (Fig. 12e–f, n = 4–6, * *p* = 0.03, unpaired *t*-test). The average band intensity for Dok7 expression was lower in MND muscle cultures compared to controls but this difference was not statistically significant (Fig. 12a–d, n = 5–6, *p* = 0.096, unpaired *t*-test). These alterations in the relative abundance of MuSK, LRP4, and Caveolin-3 proteins might influence the efficiency of the *n*-agrin-MuSK signaling system.

## Discussion

In this study we show that NMJs sampled from MND patient muscle biopsies display signs of disassembly, including diffuse AChRs and reduced localization of MuSK at the motor endplate. Myotubes generated from MND muscle did not grow large AChR clusters when co-cultured with hESC-derived motor neurons. They also failed to respond to *n*-agrin and displayed altered patterns of expression of some key proteins of the postsynaptic MuSK signaling pathway. Our results suggest that MND muscle may have defects in the *n*-agrin-LRP4-MuSK signaling pathway that may contribute to the instability of nerve-muscle connections.

Withdrawal of the motor nerve terminal and loss of postsynaptic AChR clusters are early pathological events in MND patients with lower motor neuron involvement [4, 7, 27, 45, 63]. Our examinations of NMJs from MND muscle confirm these previous observations, with evidence of motor nerve terminal sprouting, partially-denervated motor endplates, and possible dispersal of postsynaptic AChRs when compared to NMJs in non-MND muscle. Consistent with previous studies, we also found instances of motor nerve terminal withdrawal and limited terminal Schwann cell invasion [4, 45, 64], as well as muscle fiber atrophy and fiber type I grouping, which are thought to result from muscle fiber denervation followed by reinnervation by surviving motor neurons [65, 66]. These findings suggest impairment of the physiological systems that maintain the healthy nerve-muscle relationship.

Immunolabelling of MND muscle biopsies suggests that MuSK was less concentrated at the motor endplate, when compared to endplates from non-MND muscle biopsies. The subsynaptic localization of MuSK mRNA is regulated by neural activity [67]. Following surgical denervation, MuSK expression is upregulated throughout the muscle fiber, but becomes restricted to the motor endplate again after reinnervation [68]. Thus, it is possible that the relatively diffuse MuSK immunostaining observed around endplates in MND muscles reflects denervation or impaired neuromuscular transmission [69]. Nevertheless, this finding prompted us to examine the efficacy of *n*-agrin-MuSK signaling in vitro.

Muscle cells cultured from MND muscle appeared unresponsive to motor axons and *n*-agrin. By contrast, muscle cells cultured from non-MND control individuals formed large AChR clusters in response to both co-culture with human motor axons and recombinant *n*-agrin. Impaired *n*-agrin-induced AChR clustering was observed in muscle cultures derived from both sporadic MND patients (7) and from familial MND donors (2), suggesting that it might be a common phenomenon in MND muscle. While our findings are certainly indicative of a failure of the normal mechanisms by which motor axons and *n*-agrin induce and/or stabilize the postsynaptic differentiation in skeletal muscle cells, the precise nature of this failure remains uncertain.

Agrin, MuSK, LRP4, caveolin-3, and Dok7 are proteins that play key roles in driving the growth and stability of postsynaptic AChR clusters. Mouse embryos lacking agrin [9], MuSK [61], Dok 7 [70, 71] or LRP4 [72] fail to form large AChR clusters. Likewise, primary myotube cultures derived from mouse embryos lacking MuSK, LRP4, or caveolin-3 failed to cluster AChRs in response to *n*-agrin [12, 13, 16, 72, 73]. Immunoblotting revealed increased expression of MuSK and caveolin-3 in muscle cultures from MND patients, compared to those from non-MND muscle donors. The upregulation of these muscle-specific proteins is particularly striking, given the relatively slow maturation of the MND muscle cells by other measures. An upregulation of MuSK might be expected to enhance MuSK-mediated AChR clustering, except that expression of LRP4 was reduced by approximately three-fold in MND muscle cultures, compared to non-MND controls. LRP4 is the true receptor for *n*-agrin, forming a complex with MuSK to activate the tyrosine kinase pathway [10, 12, 13]. Without LRP4, *n*-agrin cannot induce AChR clustering [12, 13]. Thus, in MND muscle, a paucity of LRP4, relative to MuSK, might explain the failure of *n*-agrin-induced AChR clustering observed in our MND muscle cultures. Incipient motor endplates form in mouse embryos lacking LRP4, but they are poorly innervated and unstable [74]. Indeed, LRP4 plays an additional essential role as an inducer of presynaptic differentiation [11]. Thus, low levels of LRP4 in the specialized postsynaptic membrane might hinder reformation and maintenance of specialized presynaptic nerve terminals at the endplate in MND. If these ideas are true, then one might expect if LRP4 was elevated in MND muscle, it would slow or attenuate the withdrawal of the motor nerve terminal – such ideas remain to be tested.

Conceivably, the elevated expression of caveolin-3 might also hinder AChR cluster growth in MND muscle. Caveolin-3 can facilitate the clustering of AChRs via its interactions with MuSK [16], but overexpression of caveolin-3 causes NMJ disruption, characterized by poor AChR clusters and a downregulation of β-dystroglycan [75, 76]. Beta-dystroglycan is normally concentrated in the post-synaptic membrane. It binds to rapsyn and is important for stabilizing AChR clusters [77]. In summary, low expression of LRP4 and high expression of caveolin-3 in MND muscle cultures might both contribute to the failure of *n*-agrin-induced postsynaptic AChR clustering.

Is it possible that the MND-associated abnormalities in AChR clustering are simply a secondary consequence of denervation-reinnervation of muscle that occurs in MND patients. We think this unlikely. First, the myotubes we used in our motor neuron-muscle assays (Figs. 5, 6) and in our *n*-agrin assays (Figs. 10, 11) were generated in vitro from the progeny of muscle satellite cells sourced from MND and non-MND biopsies. The cultured muscle cells themselves had never previously been innervated, let alone denervated. Second, we demonstrate that the non-MND derived myotube cultures did respond to motor axons and *n*-neural agrin to from large clusters of AChRs, a finding that has been shown to occur across several species of muscle satellite cells by numerous researchers [8, 11, 41, 43, 60, 61, 78–80]. Third, MND satellite cells sourced, differentiated and allowed to mature to multinucleated myotubes failed to respond to either the presence of motor axons or *n*-agrin. Our protein expression data on MND and non-MND muscle cultures (Fig. 12), show a down regulation in LRP4 and Dok7 in MND cultures compared to non-MND cultures. By contrast, LRP4 and Dok7 expression increases in acute denervated muscle [69, 81, 82]. Taken together, the simplest explanation for our observations is an intrinsic fault originating within the satellite stem cells that support renewal of MND muscle ([83], see review by [84]).

While we have used human tissue and human-derived cell culture models to investigate muscle pathology and NMJ signaling defects in MND, there are some limitations to our study. Firstly, our EM analyses of MND NMJs were only qualitative, being based on a small number NMJs. Nevertheless, our ultrastructure results are consistent with previous EM analyses of NMJs from MND patients, showing flattening of the primary synaptic gutter of the postsynaptic membrane not occupied by motor nerve terminals [4, 45]. This was supported by our confocal microscopic analyses, which revealed a 50% loss of motor nerve terminal, relative to postsynaptic AChR endplate area [4, 27]. A second caveat is that our myotubes were grown from cells that were isolated from muscle biopsies. While we observed fewer and narrower myotubes in MND cultures, which is in line with previous observations of reduced differentiation capacity of MND muscle satellite cells [83], it remains uncertain whether this model system can adequately recapitulate the relevant properties of mature muscle fibers in vivo. Thirdly, while we have shown substantial differences in the expression of MuSK, caveolin-3 and LRP protein, we have yet to clarify whether these changes arise from altered patterns of gene transcription, mRNA stability, or protein stability, and whether these changes are shared among muscle from sporadic and familial MND patients. Our preliminary RNA seq data suggest that the changes in the protein expression may not be a consequence of altered levels of their transcripts, but further in-depth experiments will be required to test this relationship.

In summary, our study has revealed that muscle cell cultures derived from MND biopsies failed to form large clusters of AChRs in response to either human motor axons or *n*-agrin. Our observations support the idea that the poor responsiveness of MND myotubes is due to abnormalities in the *n*-agrin-LRP4-MuSK signaling pathway. Future studies extended to a much larger cohort of MND patients could determine what drives alterations in the expression of LRP4, MuSK and caveolin-3.

## Supplementary Information

**Additional file 1** Contains Online Resource 1 - Supplementary Methods, and Online Resource 2 - Supplementary Tables (S1 to S4) and Supplementary Figrues (S1 to S7).

## Data Availability

Single-nuclei sequencing data is available on GitHub at https://github.com/Cadaei-Yuvxvs/snRNAseq_muscle_MND.
