# Supplementary material for: Impaired signaling for neuromuscular synaptic maintenance is a feature of Motor Neuron Disease
Source: Acta Neuropathol Commun. 2022 Apr 25;10:61. doi: 10.1186/s40478-022-01360-5 (PMC9040261; doi:10.1186/s40478-022-01360-5)
Supplement: Supplementary file 1 — Additional file 1 Contains Online Resource 1 - Supplementary Methods, and Online Resource 2 - Supplementary Tables (S1 to S4) and Supplementary Figrues (S1 to S7). [file 40478_2022_1360_MOESM1_ESM.docx]

**Online Resource 1 – Supplementary Methods.**

**Muscle biopsy procedures**

*Surgical biopsies.* The area of muscle interest was identified electrically and marked pre-operatively. Local anaesthetic (bupivacaine 0.5% with adrenaline 1: 200 000) was infiltrated into the skin and subcutaneous tissues overlying the *vastus lateralis*, taking care to avoid infiltrating into the muscle itself. Additional analgesia and light, short-acting procedural sedation were also provided, at the clinical discretion of the attending neurosurgeon and anaesthetist. The anaesthetist was in attendance throughout the procedure. After incision of skin, fascia and epimysium, a block of contiguous muscle fibers (~30 x 10mm), was dissected out. After collection of the muscle sample, the wound was closed in layers with absorbable sutures.

*Needle biopsies.* At the muscle collection site, 1 mL of 1% lignocaine was injected to numb the skin and underlying fat and muscle tissue. A small (<10 mm) incision was made, and advanced through the fascia of the muscle. A 200 mg sample of muscle was collected using a sterile 6 mm hollow Bergstrom biopsy needle that had been modified for suction [1] . After the biopsy was taken, steri-strips and a compression dressing were applied to minimize any risk of further bleeding and infection. Muscle biopsies (surgical and or needle) were placed in DMEM/F-12 with 0.5% gentamicin and transferred to the laboratory for explant culture and to conduct muscle histology.

**Electron microscopy**

At the time of muscle collection, a 1 mm diameter bundle of muscle fibers was teased away from the biopsy, and immersion fixed in 2.5% glutaraldehyde plus 4% PFA in PBS for at least 12 hrs. After washing in PBS, they were transferred into 0.1 M sodium cacodylate buffer and post fixed in 2% osmium tetroxide in 0.1 M sodium cacodylate buffer. After dehydration through acetone, each block was embedded and polymerized in Epon epoxy Resin at 60^0^C for 24 hrs. Ultrathin sections (T/S) were cut at ~60-70 nm thickness, mounted onto copper grids and stained with uranyl acetate, followed by Reynolds lead citrate [2, 3]. Muscle sections were viewed and imaged using a JEOL 1010 and/or a Hitachi HT7700 transmission electron microscope.

**H9-hESC motor neuron differentiation**

Following the protocol established by Du and colleagues, the conditioned media was switched into a small molecule defined neural medium [4]. At day 13, the cells were dissociated with dispase and cultured on low adherence plates as a suspension with the same neural medium supplemented with 0.5 μM RA (Retinoic acid, Sigma-Aldrich, R2625) and 0.1 μM Purmorphamine (Santa Cruz, CA USA, SCZSC-202785). On day 19, the cells were dissociated with Accumax (Invitrogen, MA USA, 00-4666) onto a Matrigel-coated plate with the same neural medium plus 0.5 μM RA, 0.1 μM Purmorphamine and 0.1 μM Compound E (Calbiochem, Merck, Australia, 530509), and allowed to differentiate over the next 10 days.

*Defined neural medium* was composed of DMEM/F12 and Neurobasal medium at 1:1, plus 0.5X B27 (ThermoFisher, 17504-044), N2 supplement (ThermoFisher, 17502-048), Glutamax (Life Technologies, 35050-061), 0.1 mM ascorbic acid (Santa Cruz, CA USA, A45544), 3 μM CHIR99021 (Tocris, MN USA, 4423), 2 μM DMH1 (Tocris, 4126) and 2 μM SB431542 (AdooQ, CA USA, A10826) on day 1. On day 7, the cells were dissociated with dispase at 1 mg/ml (ThermoFisher, 17105-041) and passaged with the same neural medium as day 1, supplemented with 0.1 mM ascorbic acid, 1 μM CHIR99021, 2 μM DMH1, 2 μM SB431542, 0.1 μM retinoic acid (RA; Sigma-Aldrich, R2625) and 0.5 μM Purmorphamine (Santa Cruz, SCZSC-202785).

**Immunostaining of microfluidic co-cultures**

For immunostaining of co-cultures, culture media was removed from the microfluidic device and replaced with Alexa-488 α-bungarotoxin (Invitrogen) in medium (DMEM/F12 plus 2% horse serum Gibco 16050-130) to detect AChRs. After 1 hr incubation in the cell culture incubator, the microfluidic culture was rinsed twice with PBS (5 min each) and fixed with 4% PFA in PBS for 30 min at room temperature. After rinsing 3 x 5 mins in PBS, cells were blocked with buffer containing 2% BSA and 0.1% Triton X-100 for 1 hr at room temperature. Cells were incubated with primary antibodies to neurofilament 200 and SV2 overnight at 4°C. Cells were then washed 3 x 5 min in PBS, followed by incubation with Alexa 555 goat anti-rabbit plus Alexa 647 sheep anti-mouse conjugated secondary antibodies for 2 hrs at room temperature. Hoechst 33342 (1 mg/mL; Invitrogen MA USA) was also added to this incubation to locate muscle nuclei. This incubation step was followed by 3 x 5 min washes with PBS. Cells were maintained in PBS for image acquisition. Antibody details are listed in **Table S4**.

**Image acquisition of H9-MNs, and H9-MN muscle microfluidic cultures**

Confocal images of H9-MN-muscle microfluidic cultures were captured using a Leica DMi8 SP8 Inverted Confocal microscope, using a 40X air NA 0.85 objective, at a resolution of 1024 x 1024 pixels, bidirectional scanning mode at a z-step of 0.37 μm. Laser power levels, scan speed, photomultiplier gain levels and a pinhole size of 1 were the same across all cultures. All microfluidic cultures were imaged using the excitation diode laser lines 473 nm, 559 nm, 635 nm. We also included the transmitted light detector (TLD) channel as a pseudo phase image capture of the myotubes and the Hoechst nuclei housed within their cytoplasm. Images were reconstructed and analyzed using Imaris imaging software (Bitmap). This involved the surface rendering of neurofilament and SV2 stained motor axons, the phase surface of adjacent myotubes and their Alexa-488 stained AChR cluster patches. Total AChR area was normalized to total neurofilament area per visual field (400 μm^2^ X 400 μm^2^; as per [5]). Between 4 to 13 visual fields per culture were selected and analyzed. The criteria used for identifying a myotube for AChR analyses is described above. The identity of the muscle in the muscle chamber was withheld from the investigator until all analyses were complete.

**Bioassays for n-agrin clustering co-staining for Desmin**

Post labelling for AChRs with Alexa-555 α-bungarotoxin, muscle cultures were then washed with PBS then fixed with methanol (-20°C) for 3 min. Following fixation, coverslips were rinsed with PBS then blocked with 2% BSA and permeabilised with 0.1% Triton X-100 in PBS for 30 min at room temperature. Coverslips were incubated overnight at 4^o^C with a desmin primary antibody in 0.1% Triton X-100 in PBS. Coverslips were rinsed with PBS then incubated for 1 hr with Alexa-555 goat anti-rabbit. Coverslips were washed with PBS and mounted onto Superfrost™ slides with Mowiol (Sigma Aldrich).

**Immunostaining of muscle biopsies**

For wholemount immunostaining, teased muscle bundles were incubated with Alexa-488 conjugated α-bungarotoxin overnight at 4^o^C. They were then washed in PBS, then blocked in 4% BSA in 0.5% TritonX-100 in PBS for 4 hrs at 4^o^C. Next, muscle fibers were incubated overnight at 4^o^C in a cocktail of rabbit anti-neurofilament 200 plus rabbit synaptophysin. The next day, muscle fibers were washed in 1% BSA in 0.25% Triton X-100 for 12 hrs at 4^o^C, prior to an overnight 4^o^C incubation with Alexa-555 goat anti-rabbit. Muscle fibers were washed in PBS over the course of the following day, prior to mounting in Fluorogold antifade medium (Bio-Rad, USA).

Cryo-sectioned muscles were air dried and then blocked with 2% BSA and 0.1% TX-100 for 1 hr at room temperature. These sections were then incubated overnight with either sheep or rabbit anti-MuSK at 4^o^C. Next, they were washed with PBS and incubated with a combination of Alexa-555 α-bungarotoxin (Invitrogen) and an Alexa-488 secondary goat anti-rabbit (Invitrogen) for 2 hrs at room temperature. Sections were then rinsed with PBS, mounted with Prolong gold antifade reagent (Invitrogen). Sections were imaged with an Olympus Fluoview FV1000 confocal laser scanning microscope, equipped with two excitation diode laser lines (473 nm and 559 nm) running on Fluoview FV10-ASW software version 01.07C. Images were taken at a resolution of 1024 by 1024 pixels, using a 100x/ 1.35 NA Oil Iris UPlan-Apochromat objective with a Z-step size of 0.3 μm. All images labeled with the same antibody were captured using identical laser power levels, photomultiplier gain levels, scanning speed and pinhole size.

**Image acquisition and analyses of intact NMJs**

Digital images of NMJs from either muscle whole mounts or sections were acquired using a Leica DMi8 SP8 inverted confocal microscope equipped with excitation diode laser lines (473 and 559 nm), running Leica LAS X software (Leica Microsystems, Wetzla, Germany). Images were taken at a resolution of 1024 x 1024 pixels using a 63x glycerol objective (NA 1.4), scanning speed of 400, bidirectional scanning mode at a z-step of 0.37 μm. Sequential scanning of the different channels was performed to limit fluorphore spectral cross talk. Laser power levels, scan speed, photomultiplier gain levels and a pinhole size of 1 were maintained at same level across the different immunostained wholemount preparations, and across the different immunostained muscle sections. Saved images were reconstructed and analysed using Fiji imaging software [6]. The innervation status of these intact NMJs was classified using the following criteria: **i**) *innervated* *NMJs*- motor nerve terminal covering ~60% of all the AChR patches that make up the endplate; **ii**) *partially denervated-reinnervated* *NMJs*– nerve staining covering less than 50% of AChR patches and/or with evidence of one or more AChR patches having no pre-synaptic staining and/or evidence of motor axonal sprouting.

For NMJ morphometry, maximum Z-projections were analyzed. Fluorescent channels were selected to reveal either AChRs or motor axons and their terminal endings. The free hand drawing tool was used to draw around individual AChR patches of an individual NMJ, or the opposing motor nerve terminal ending. NMJs were defined by their location on one identified muscle fiber. Quantification of the thickness of the terminal motor axon was determined by placing the Fiji line scan tool at right angles to the motor axon at a set point; namely the primary incoming motor axon ~2-3 μm before it branched into its final terminal axons was measured. Terminal axon thickness was also measured in same manner ~2 μm past the final primary axon branch.

**Isolation of single nuclei from myotubes for RNA sequencing**

Each 10 cm dish of cultured muscle cells was washed twice with PBS. Cells were then enzymatically dissociated with 1.5 mL of TryPLE Express (Gibco) for 3-5 min at 37°C, 5% CO_2_. TryPLE was neutralized with 1.5 mL of differentiation medium; this TryPLE/differentiation medium solution was then collected into a 15 mL polypropylene tube. Next, dishes were washed 3 times with 1mL of differentiation media, with each wash being collected into the 15 mL tube. Remaining cells were removed using a cell scraper and transferred into the 15 mL tube. Cells underwent a final 1mL PBS wash, and this PBS was also collected into the 15 mL tube. For each sample, suspended cells were first passed through a 100 µm strainer into a new 15 mL polypropylene tube. Samples were transferred onto ice. Samples were spun at 1000xg for 5 min (4°C), then transferred back onto ice for media aspiration. Cold nuclear extraction buffer (1 mL; 320 mM Sucrose (Sigma Aldrich), 10 mM Tris-HCL (pH 8), 5 mM CaCl2, 3 mM Mg (acetate), 2 0.1 mM EDTA, 1 mM DTT, 10 mM NaCl, 0.3% IGEPAL, 1x Merck Protease Inhibitor Set III, 172 ng/µl RNAsin, Autoclaved MilliQ water) was added to each cell pellet and gently resuspended. Next, cell suspensions were filtered through a 70µm strainer back into the primary 15 mL tube and incubated on ice for 2-3 min. After this time, 1 mL of each sample was transferred through a 40 µm cell strainer into a Dounce homogeniser on ice. Following homogenization, cell suspensions were transferred through a 40 µm cell strainer into a new 15 mL polypropylene tube on ice. Samples were then centrifuged at 3000xg for 7 min at 4°C, and supernatant was aspirated. 50 µl of cold PBS and 172 ng/µl RNAsin was added onto cell pellets and incubated on ice for 15 min. Sample volume was increased to 1mL with PBS and RNAsin and DAPI (0.1 µg/ml), gently resuspended, and transferred into a FACS tube (pre-cooled on ice) through a 40µm cell strainer. Cells were FACS sorted to collect DAPI positive nuclei, and sequencing was performed using the Chromium system from 10X Genomics (10X Genomics, Pleasanton, CA, USA).

**Statistical analyses of RNA Seq Data**

First, droplets with zero unique molecular identifier counts (droplets presumably lacking a nucleus) were removed from the raw count matrices. In addition, droplets with less than 5% of genes expressed and/or more than 5% of counts mapped to mitochondrial genes were filtered from the count matrices. The count matrices were log-normalized using the R package, Seurat (version 4) [7]. The R package, DoubletFinder [8], was then used to identify and remove droplets that contained the RNA content of two nuclei, which would be a technical artifact. Using Seurat’s standard integration pipeline, all four muscle cultures were normalised for batch-effect, such that technical variations are removed and only biological variation is preserved [7]. Further, we used Seurat to predict cell types by projecting a reference atlas on our data [9]. For the reference atlas, we used a manually annotated single-cell dataset of human skeletal muscle samples taken from various anatomical sites [10]. The R package, Clustree [11] was used to determine the optimal resolution for Louvain clustering [12] [13]. The optimal resolution was chosen based on the highest average stability score. To visualize the data, we dimensionally reduced the data with principal component analysis then visualized the first 20 principal components on a 2D UMAP plot [8]. We used the R package, MAST [14] to identify marker genes by performing differential expression analysis between groups of interest.

**References**

1. Tarnopolsky, M.A., et al., *Suction-modified Bergström muscle biopsy technique: experience with 13,500 procedures.* Muscle Nerve 2011. **43**: p. 717-725.

2. Banks, G.B., et al., *Glycinergic and GABAergic synaptic activity differentially regulate motoneuron survival and skeletal muscle innervation.* J Neurosci, 2005. **25**(5): p. 1249-59.

3. Noakes, P.G., et al., *Aberrant differentiation of neuromuscular junctions in mice lacking s-laminin/laminin beta 2.* Nature, 1995. **374**(6519): p. 258-62.

4. Du, Z.-W., et al., *Generation and expansion of highly pure motor neuron progenitors from human pluripotent stem cells.* Nat Commun, 2015. **6**: p. 6626.

5. Yoshida, M., et al., *Modeling the early phenotype at the neuromuscular junction of spinal muscular atrophy using patient-derived iPSCs.* Stem Cell Reports, 2015. **4**(4): p. 516-568.

6. Schindelin, J., et al., *Fiji: an open-source platform for biological-image analysis.* Nat. Methods, 2012. **9**(7): p. 676-682.

7. Hao, Y., et al., *Integrated analysis of multimodal single-cell data.* Cell, 2021. **184** (13): p. 3573-3587.

8. McInnes, L., J. Healy, and J. Melville, *Umap: Uniform manifold approximation and projection for dimension reduction.* arXiv, 2020. **arxiv.org**: p. arXiv:1802.03426v3.

9. Stuart, T., et al., *Comprehensive integration of single-cell data.* Cell, 2019. **177**(7): p. 1888–1902.e21.

10. De Micheli, A.J., et al., *A reference single-cell transcriptomic atlas of human skeletal muscle tissue reveals bifurcated muscle stem cell populations.* Skeletal Muscle 2020. **10:19**: p. 2-13.

11. Zappia, L. and A. OShlack, *Clustering trees: a visualization for evaluating clusterings at multiple resolutions.* Gigascience., 2018. **7**(7): p. giy083.

12. Subelj, L. and M. Bajec, *Unfolding communities in large complex networks: combining defensive and offensive label propagation for core extraction.* Phys Rev E Stat Nonlin Soft Matter Phys., 2011. **83**(2): p. 036103.

13. Blondel, V.D., et al., *Fast unfolding of communities in large networks. Journal of statistical mechanics: theory and experiment. .* Journal of statistical mechanics: theory and experiment, 2008. **2008**(10): p. P10008.

14. Finak, G., et al., *MAST: a flexible statistical framework for assessing transcriptional changes and characterizing heterogeneity in single-cell RNA sequencing data.* Genome Biol. , 2015. **16**: p. 278.

15. Ammar, A.B., et al., *A Mutation Causes MuSK Reduced Sensitivity to Agrinand Congenital Myasthenia.* PLOS ONE, 2013. **8**: p. e53826.

16. Cole, R.N., et al., *Patient autoantibodies deplete postsynaptic muscle-specific kinase leading to disassembly of the ACh receptor scaffold and myasthenia gravis in mice.* J Physiol. , 2010. **588**(Pt 17): p. 3217-3229.

17. Choi, H.Y., et al., *APP interacts with LRP4 and agrin to coordinate the development of the neuromuscular junction in mice.* eLife, 2013. **2**: p. e00220.

**Online Resource 2 - Supplementary Tables and Figures**

**Supplementary Tables**

**Supplementary Table S1**. Percentage of Type I and Type II muscle fibres.

| **Donor ID** | **Type 1** | **Type 2** | **Total Fibre/500um^2^** | **% Type I** | **% Type II** |
| --- | --- | --- | --- | --- | --- |
| Con-3^#^ | 13 | 46 | 59 | 22% | 78% |
| Con-5* | 32 | 41 | 73 | 44% | 56% |
| Con-4* | 17 | 23 | 40 | 42% | 58% |
|  |  |  |  |  |  |
| MND-1* | 44 | 41 | 85 | 58% | 48% |
| MND-3* | 44 | 14 | 58 | 76% | 24% |
| MND-4* | 36 | 21 | 57 | 63% | 37% |
| MND-5* | 31 | 28 | 59 | 53% | 47% |
| MND-6* | 34 | 33 | 67 | 50% | 50% |
| MND-7* | 17 | 21 | 38 | 45% | 55% |

**Vastus Lateralis* muscle and ^#^*Deltoid* muscle.

**Supplementary Table S2.** NMJ Morphology for individual donor biopsies

| **Donor ID and disease status** | **Con-1^@^** | **Con-2^@^** | **Con-3**^#^ | **Con-4^@^** | **Con-5^@^** | **MND-2^@^** | **MND-3^@^** | **MND-4^@^** |
| --- | --- | --- | --- | --- | --- | --- | --- | --- |
| Total No. of NMJs /Donor | 2 | 12 | 19 | 22 | 12 | 13 | 40 | 24 |
| Mean No. of terminal motor axons inputs | 1 | 1 | 1 | 1 | 1 | 1 | 1 | 1 |
| Mean primary Motor Axon thickness μm | 1.9 +/-1.3 | 1.03  +/-0.5 | 1.5 +/-0.6 | 1.2+/- 0.4 | 2.1 +/-  0.3 | 0.67  +/-0.23 | 1.1  +/-0.3 | 2.0  +/- 0.5 |
| Mean No. of secondary motor axon branches | 2.5  +/- 0.7 | 2.3  +/- 0.7 | 2.3  +/- 0.5 | 3.6  +/- 1.1 | 2.2  +/- 0.4 | 2.2  +/- 0.8 | 1.8  +/- 0.4 | 2.3  +/- 0.7 |
| Mean Secondary axon thickness μm | 0.80+/-0.1 | 0.67 +/- 0.5 | 0.94  +/-0.4 | 0.64 +/-0.3 | 1.58  +/-0.4 | 0.42  +/-0.2 | 0.66  +/-0.2 | 1.11  0.4 |
| Presence of nodal sprouts | 0/2 NMJs | 3/13  NMJs |  | 11/23  NMJs |  |  |  |  |
| Nerve terminal to AChR overlap | 65.2%  +/- 13.3 | 60.2%  +/- 5.2 | 59.2%  +/- 9.5 | 57.97%  +/- 18.2 | 58.13%  +/- 7.6 | 35.1%  +/- 4.7 | 31.8%  +/- 10.7 | 21.5%  +/- 7.3 |
| No. of AChR cluster patches per NMJ | 8.5  +/- 3.5 | 7.7  +/- 2.9 | 9  +/- 2.8 | 4.8  +/- 1/5 | 8.4  +/- 3.2 | 10.8  +/- 3.6 | 7.1  +/- 3.9 | 7.4  +/- 3.4 |
| Mean AChR cluster patch size (μm^2^) | 16.78  +/- 5.6 | 12.76  +/- 8.4 | 14.68  +/- 5.8 | 10.9  +/- 4.4 | 13.99  +/- 5.2 | 12.1  +/- 6.0 | 16.90  +/- 9.1 | 27.46  +/- 14.7 |
| Mean Total AChR cluster area /NMJ (μm^2^) | 124.8.2  +/- 81.2 | 102.2  +/- 57.8 | 137.7  +/- 33.9 | 149.1  +/- 47.6 | 118.9  +/- 45.2 | 145.1  +/- 41.4 | 108.2  +/- 61.7 | 160.7  +/- 50.2 |
| Mean No. of nerve terminal boutons/NMJ | 8.5  +/- 3.5 | 7.7  +/- 2.9 | 8.8  +/- 2.9 | 4.83  +/- 1.5 | 8.4  +/- 3.2 | 10.8  +/- 3.6 | 5.7  +/- 2.6 | 8.1  +/- 3.7 |
| Mean nerve terminal bouton size (μm^2^) | 10.22  +/- 3.6 | 7.52  +/- 4.6 | 8.9  +/- 3.9 | 5.5  +/- 4.1 | 8.0  +/- 3.2 | 4.24  +/- 2.4 | 4.9  +/- 2.7 | 5.9  +/- 4.0 |
| Total nerve terminal area (μm^2^) | 76.64  +/- 36.2 | 60.11  +/- 31 | 81.0  +/- 21.6 | 85.78  +/- 38.7 | 68.1  +/- 23.9 | 50.54  +/- 15 | 27.33  +/- 14.3 | 32.27  +/- 22.6 |

Means +/- SDs are calculated using one sample t and Wilcoxon test, assuming Gaussian distribution; ^@^*Vastus Lateralis* muscle; ^#^*Deltoid* muscle.

**Supplementary Table S3.** Primer Sequences for motor neuron genes.

| **Target Gene** | **Forward Primer Sequence** | **Reverse Primer Sequence** | **Product Size (bp)** |
| --- | --- | --- | --- |
| PAX6 | CAGCTTCACCATGGCAAATAA | ATCATAACTCCGCCCATTCA | 112 |
| OLIG2 | ATAGATCGACGCGACACCAG | ACCCCAAAATCTGGATGCGA | 90 |
| HB9 | CAGCTGGACCAGTGGCTG | ACTTCCCCAGGAGGTTCGAC | 97 |
| ISL1 | GGACAAGAAGCGAAGCATCA | CCGTCGTGTCTCTCTGGACT | 120 |
| ChAT | CCAATCGCTGGTACGACAA | AGTGTTCGCACACCACACC | 75 |
| *ETFA | TGTTGATGCTGGCTTTGTTC | TGGATGGCTCCAGATATTCC | 102 |

*ETFA house-keeping gene.

**Supplementary Table S4.** Primary and secondary antibodies used in this study.

| **Antibody** | **Species (Isotype)** | **Dilution in**  **blocking buffer** | **Supplier and Catalog Number** |
| --- | --- | --- | --- |
| Anti-Neurofilament 200 | Rabbit Polyclonal (IgG) | 1:1000**^** | Sigma-Aldrich – N4142 |
| Anti-Synaptophysin | Rabbit Polyclonal (IgG) | 1:50**^** | Dako – A0010, RRID:AB_2315411 |
| Anti-MuSK | Rabbit Polyclonal (IgG) | 1:500***** | Supplied by William Phillips |
| Anti-Islet-1 | Rabbit Polyclonal (IgG) | 1:500***** | Abcam – ab20670 |
| Anti-ChAT | Goat Polyclonal (IgG) | 1:200***** | Millipore – AB114P, RRID:AB_2079751 |
| Anti-SV2 | Mouse Monoclonal (IgG1) | 1:50* | DSHB Uni of Iowa. RRID:AB_2315387 |
| Anti-Desmin | Rabbit Monoclonal (Y66) | 1:50* | Abcam – ab32362, RRID:AB_731901 |
| Anti-Myosin heavy chain | Rabbit Polyclonal | 1:50* | Sigma Aldrich (M7658) |
| Anti-Dok7 | Goat Polyclonal | 1:1000^#^ | R & D Systems AF6398  RRID:AB_10718400 |
| Anti-MuSK 610 T | Rabbit Polyclonal | 1:1000^#^ | Kind gift of Markus Reugg, [15] |
| Anti-MuSK | Sheep Polyclonal | 1:500 | Supplied by William D. Phillips [16] |
| Anti-LRP4 | Rabbit Polyclonal | 1:1000^#^ | Kind gift of Stephan Kroger [17] |
| Anti-Myosin fast (f) | Mouse Monoclonal | 1:40 | Leica WB-MHCf (NCL-MHCf)  RRID:AB_563899 |
| Anti-Myosin slow (s) | Mouse Monoclonal | 1:80 | Lecia WB-MHCs (NCL-MHCs)  RRID:AB_563898 |
| Anti-Tubulin | Mouse Monoclonal | 1:4000^#^ | Sigma-Aldrich (T5168) |
| Alexa-555 goat anti-rabbit | Goat Polyclonal | 1:500^ or * | Invitrogen (A32732) |
| Alexa-488 donkey anti-goat | Donkey Polyclonal | 1:500^ or * | Invitrogen (A32814) |
| Alexa-555 sheep anti-rabbit | Sheep Polyclonal | 1:500^ or * | Abcam (ab150182) |
| Alexa-555 goat anti-mouse | Goat Polyclonal | 1:500^ or * | Invitrogen (A32727) |
| Alexa-647 Sheep anti-mouse | Sheep Polyclonal | 1:500^ or * | R&D systems (IC016R) |
| Alexa-488 goat anti-rabbit | Goat Polyclonal | 1:1000* | Invitrogen (A32731) |
| HRP anti-mouse | Mouse monoclonal | 1:10,000^@^ | Sigma-Aldrich (12-348) |
| HRP anti-goat | Rabbit polyclonal | 1:10,000^@^ | Sigma-Aldrich (AP106P) |

^#^5% (w/v) non-fat dried skim milk powder in 10mM Tris-HCL, pH 7.5, 150mM NaCl, and 0.1% Tween 20; *2% BSA and permeabilized with 0.1% Triton X-100 in PBS; ^4% BSA in 0.5% TritonX-100 in PBS; ^@^1% (w/v) skim milk in TBST.

**Supplementary Figures with legends**


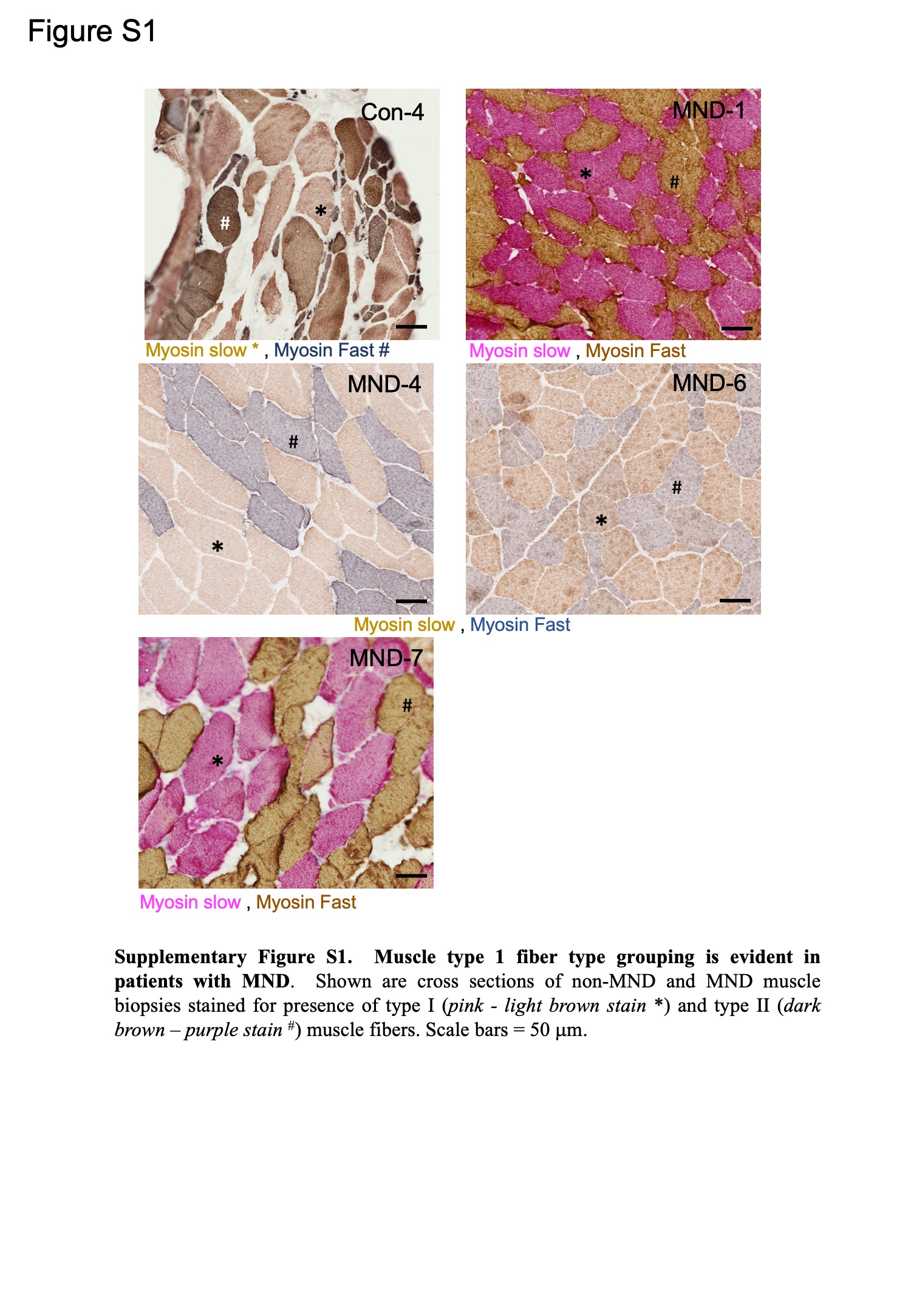


**
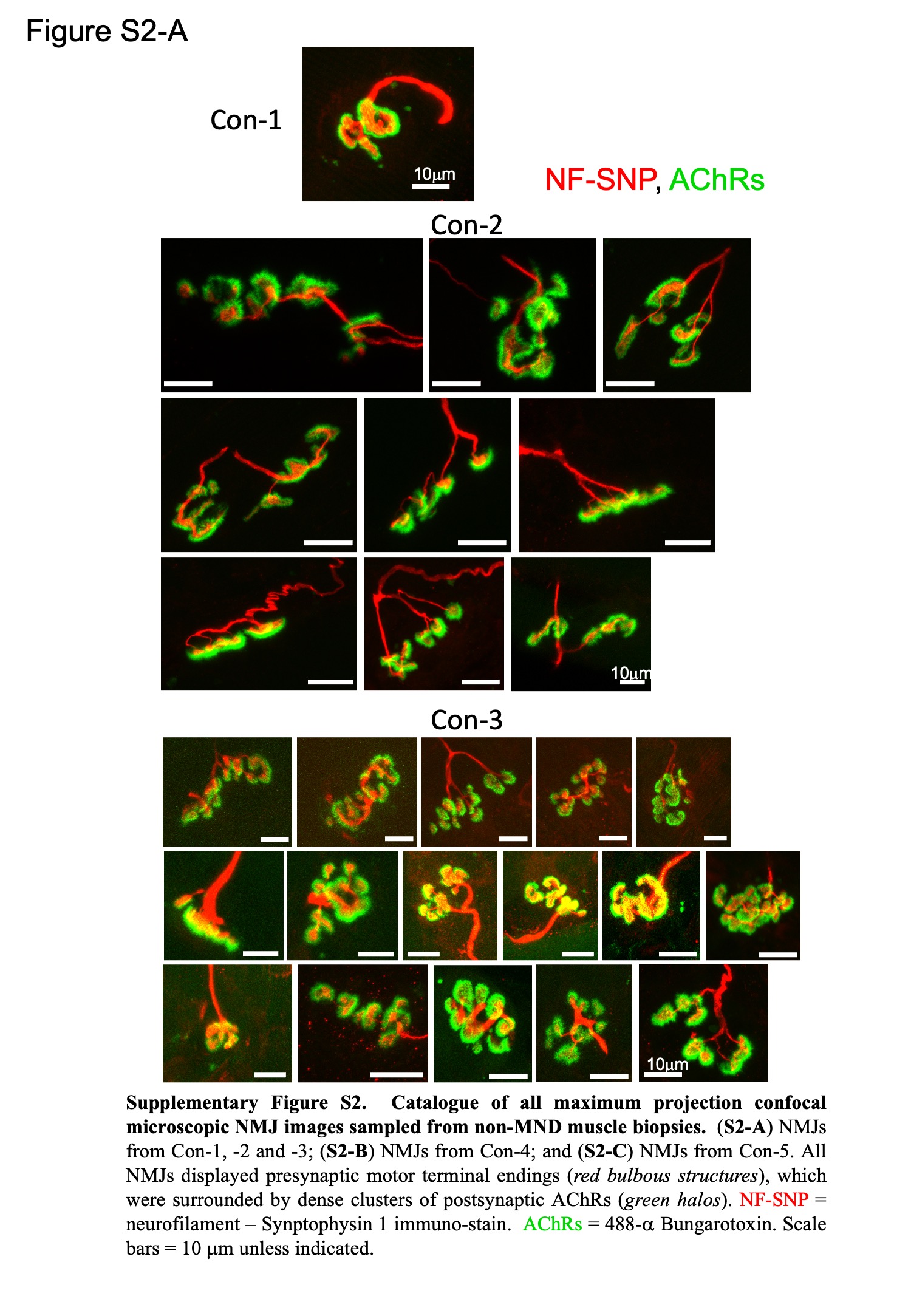
**

**
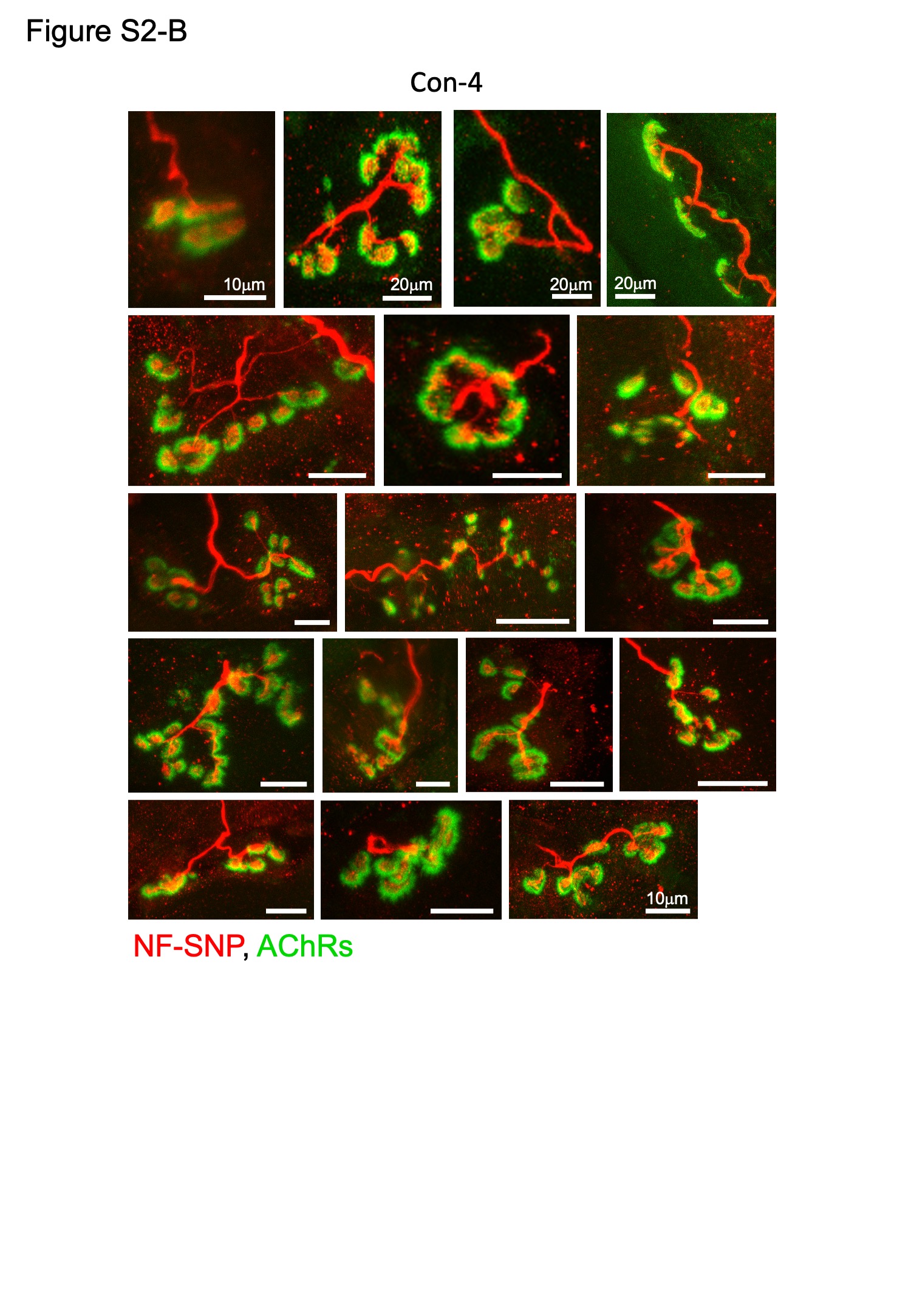
**

**
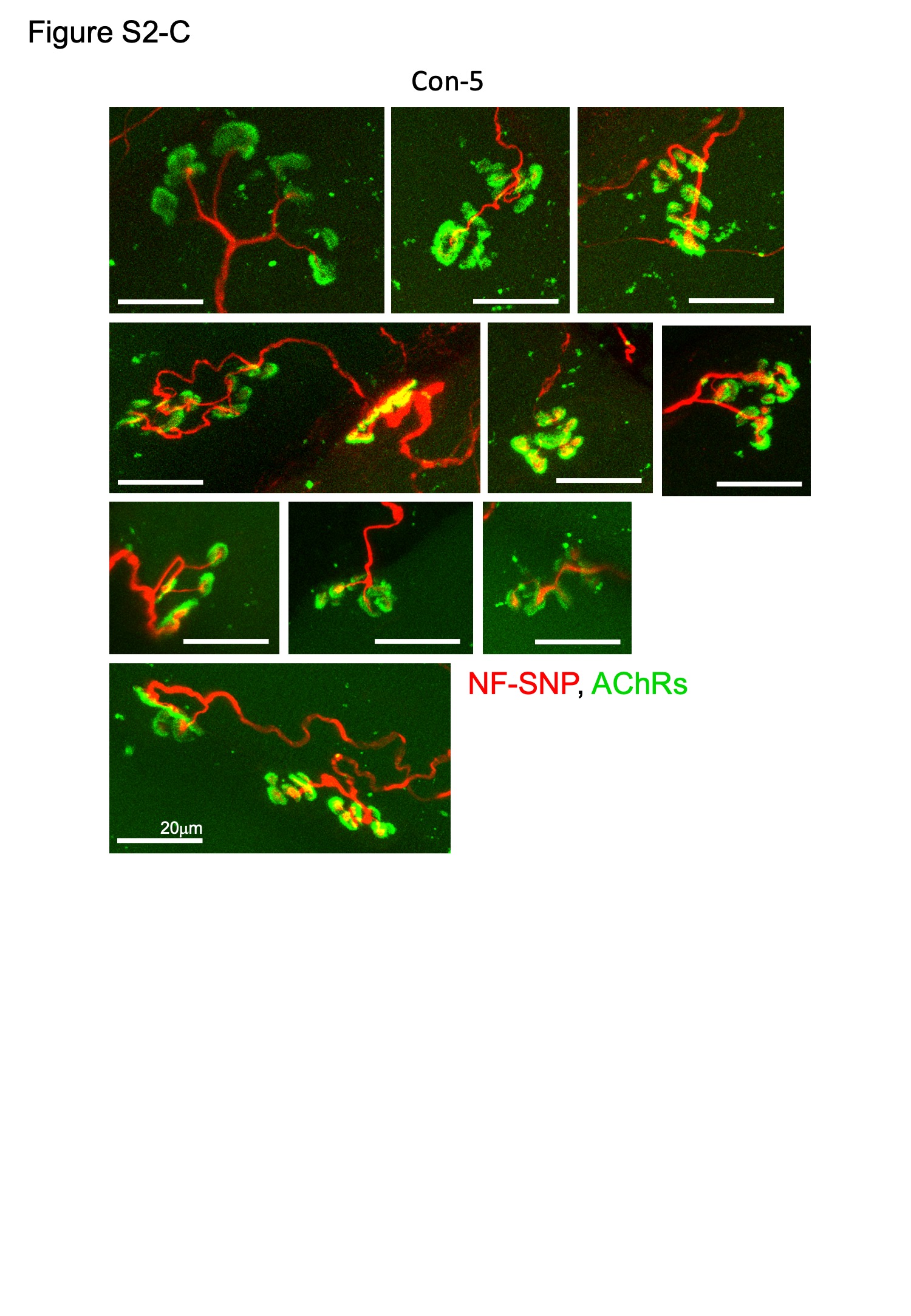
**

**
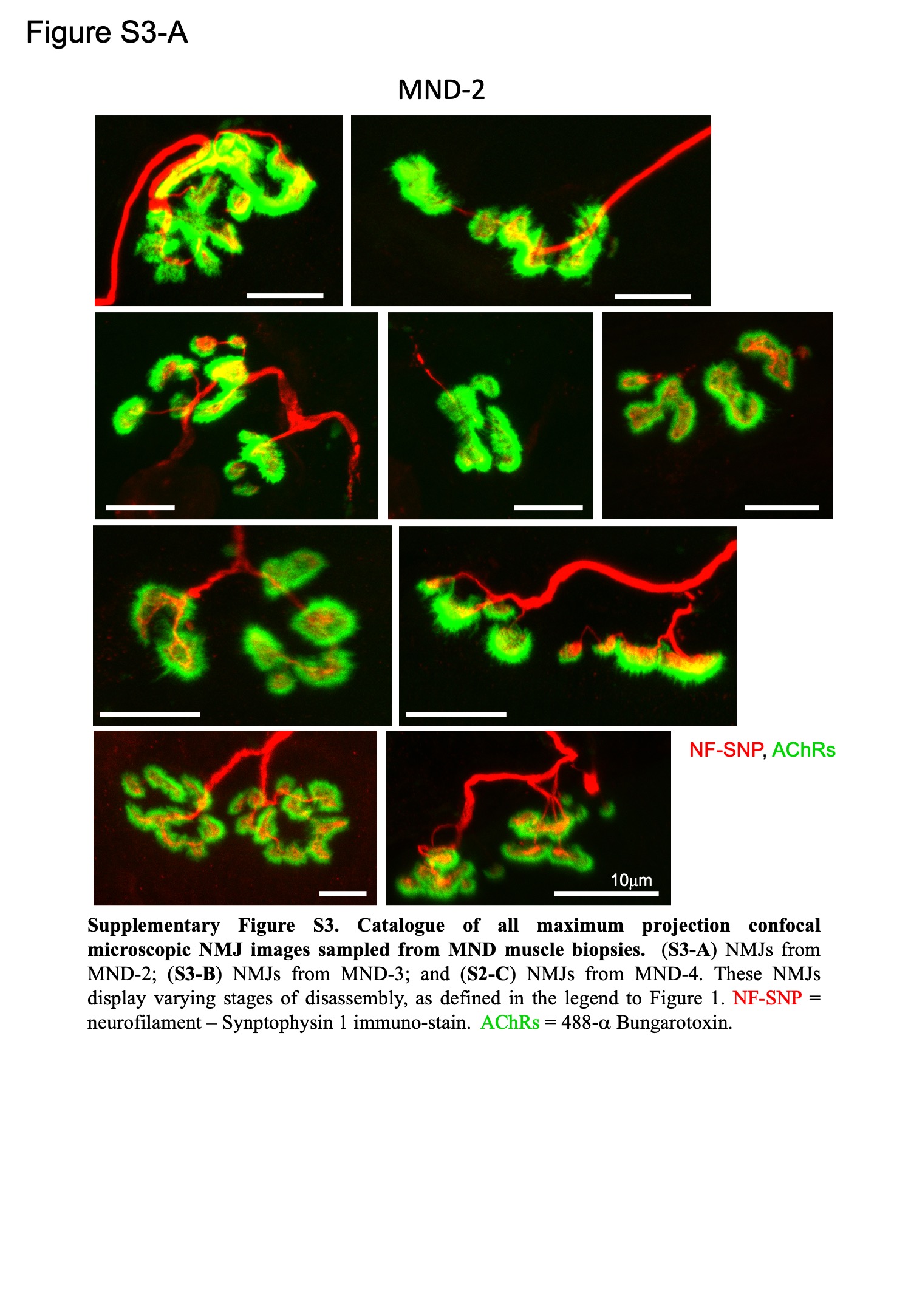
**

**
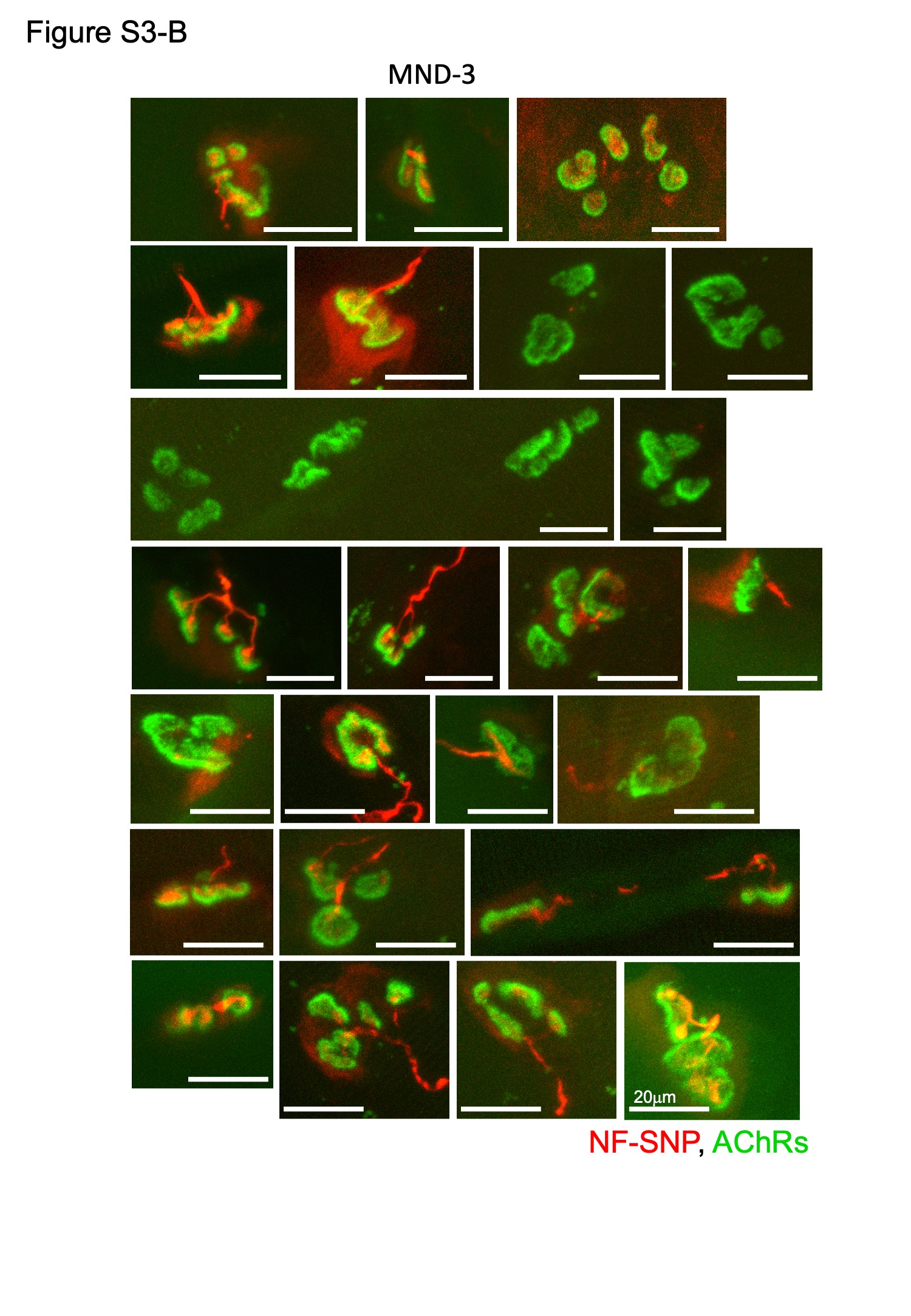
**

**
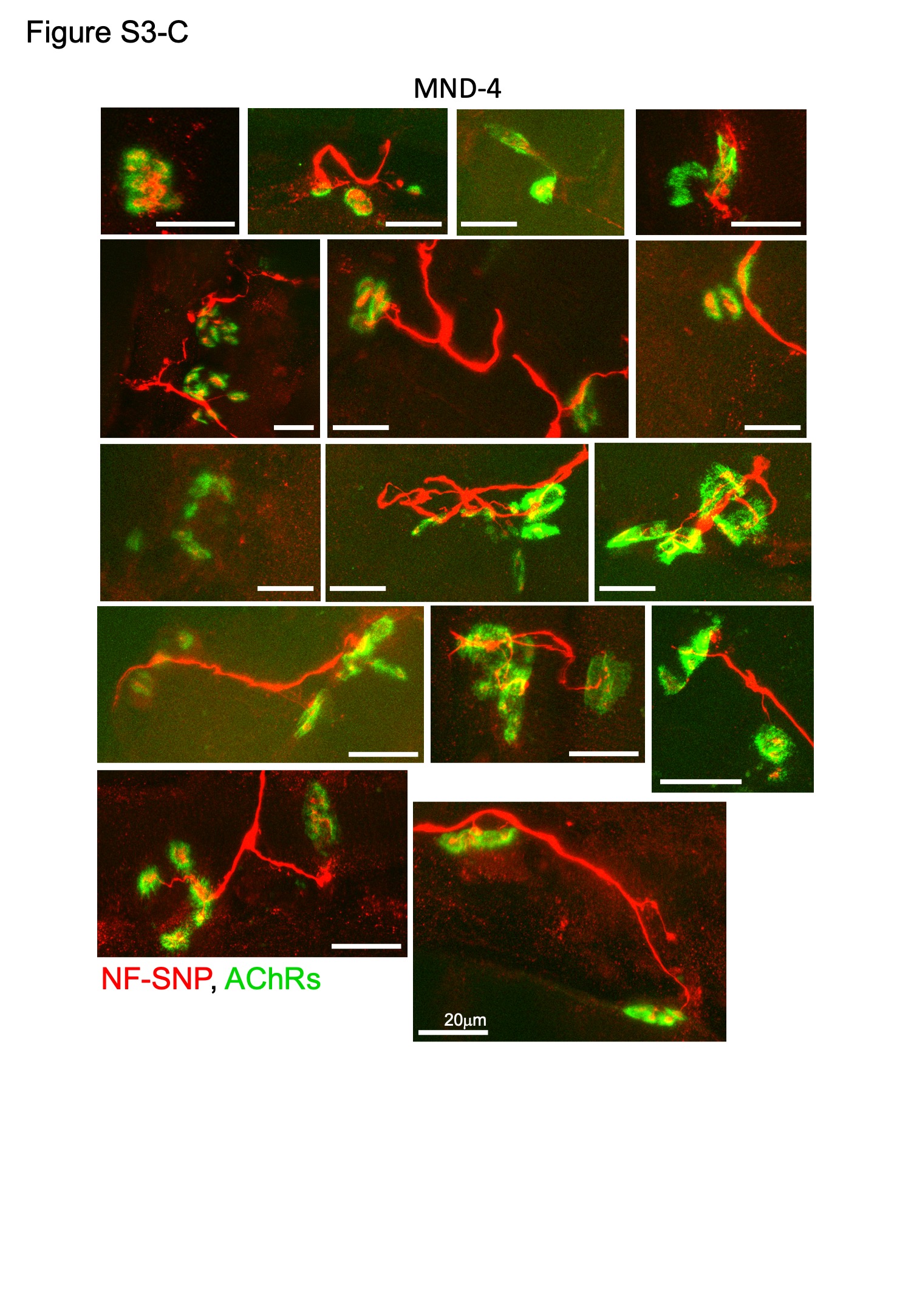

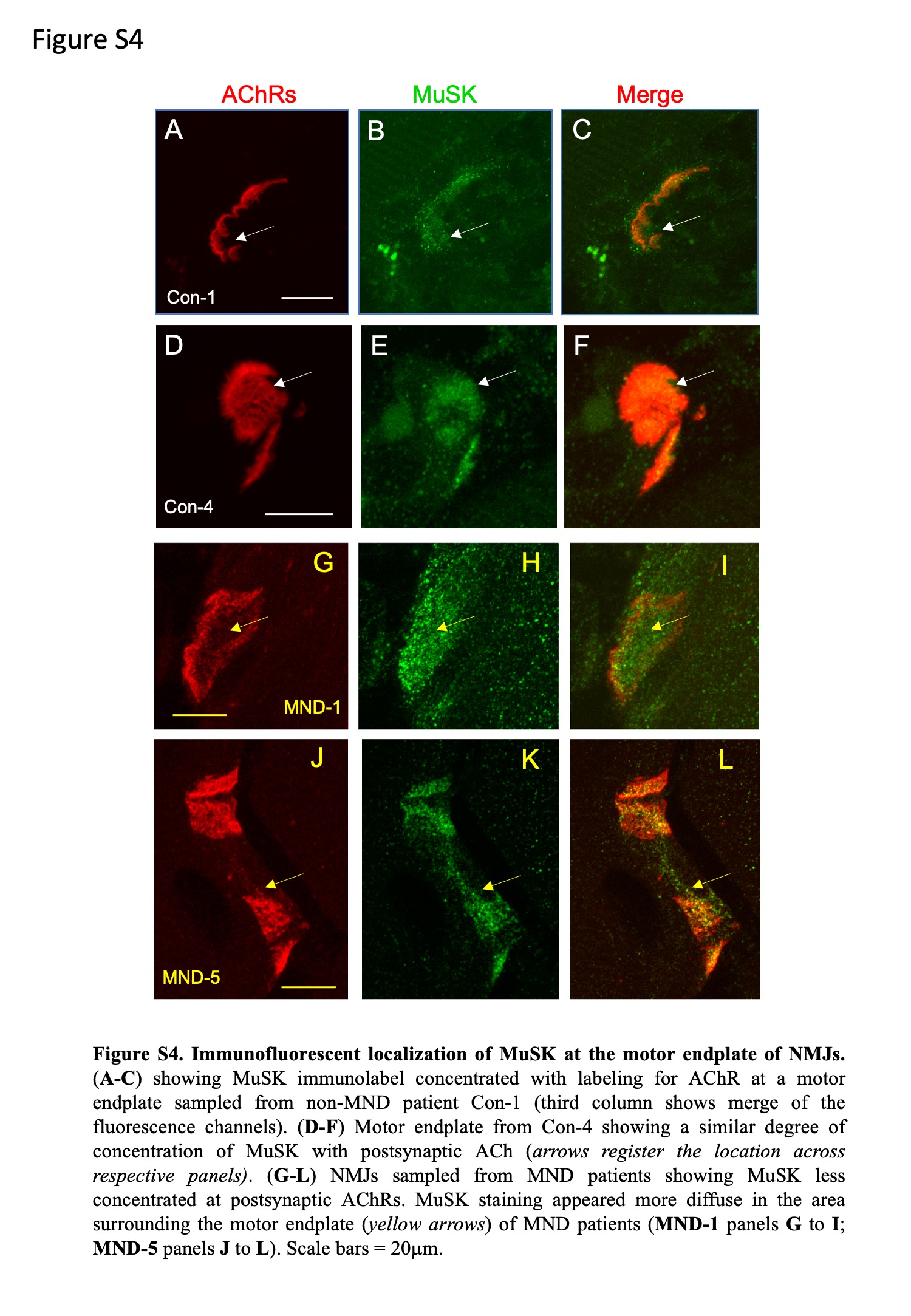
**

**
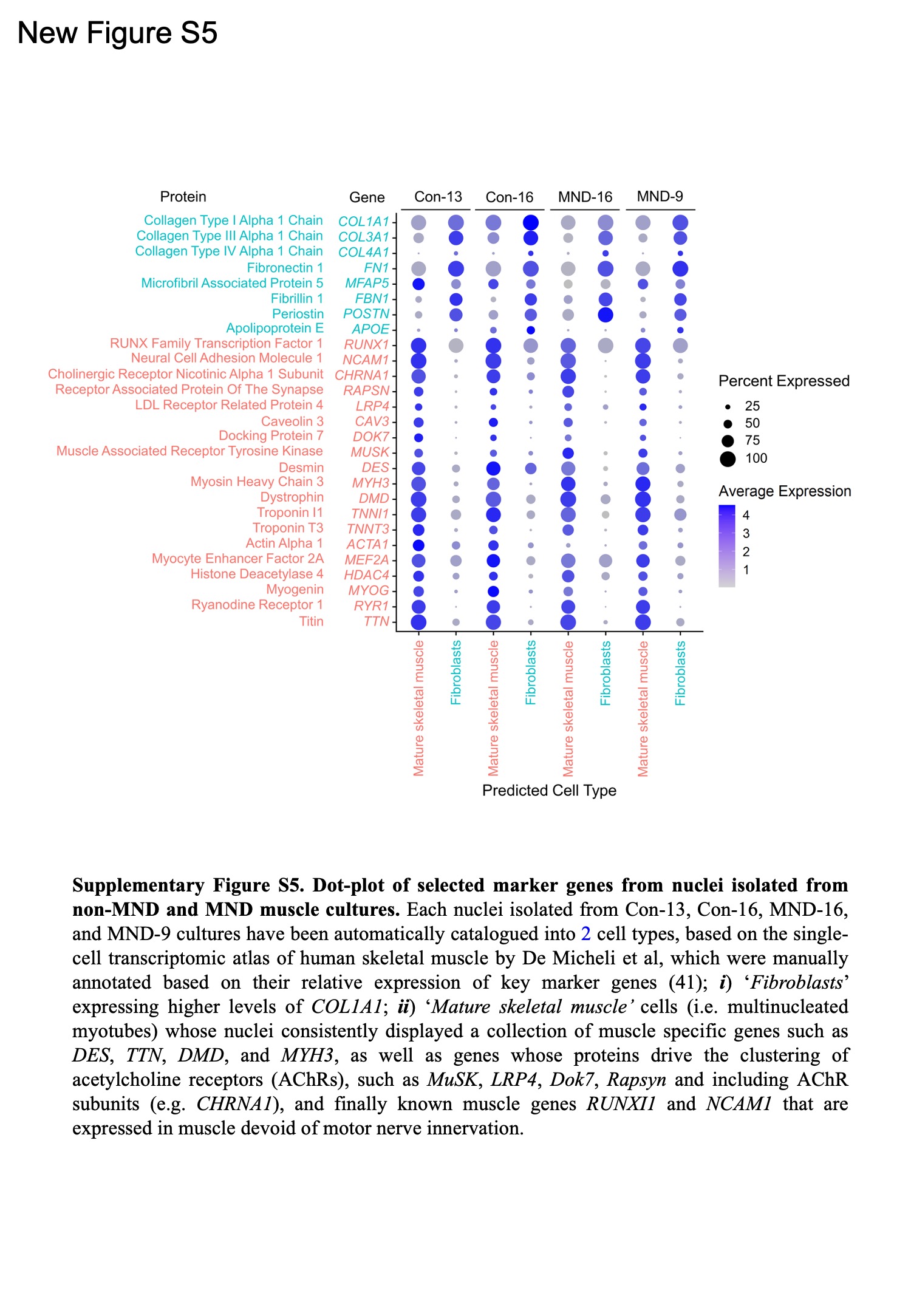
**

**
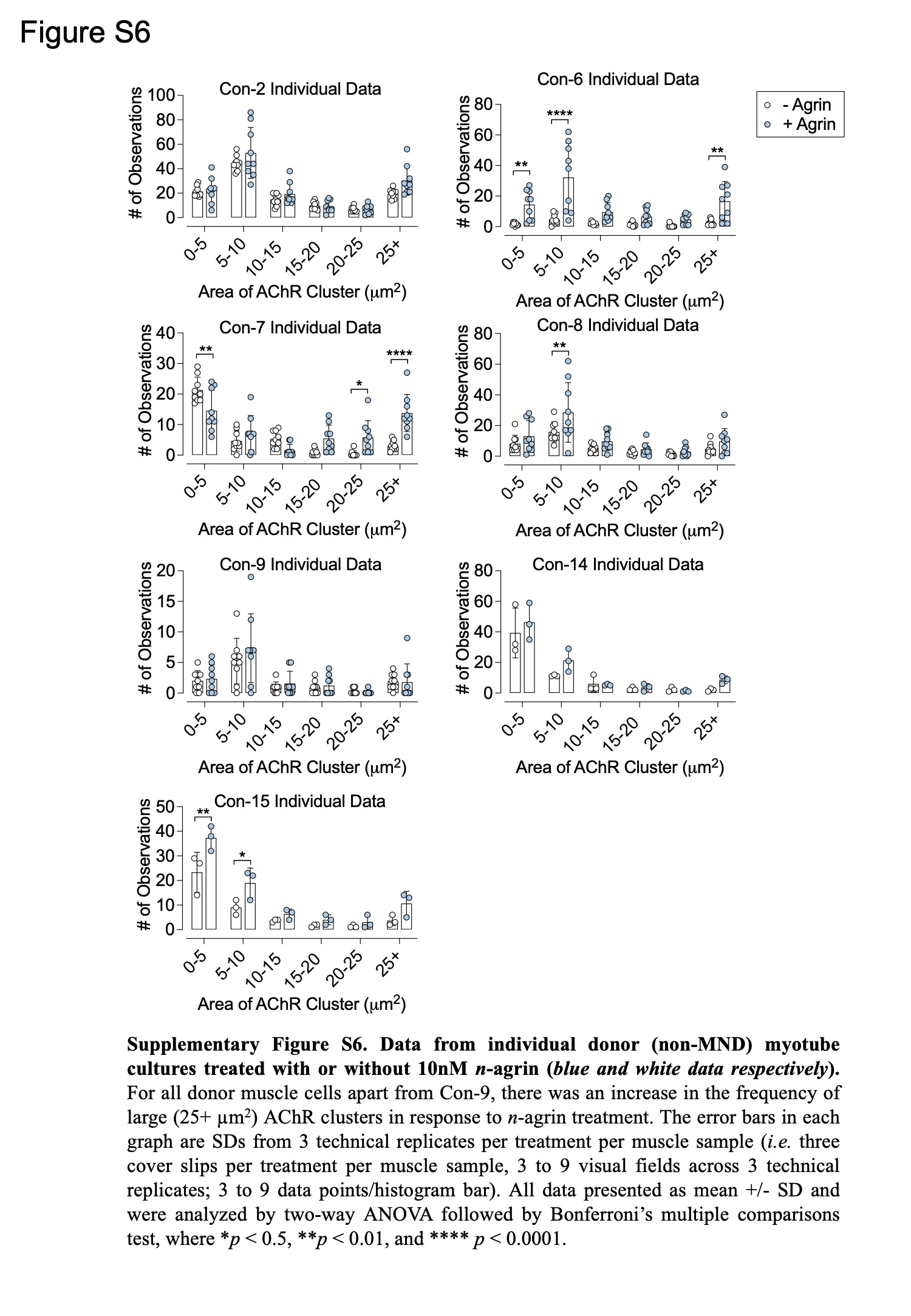
**

**
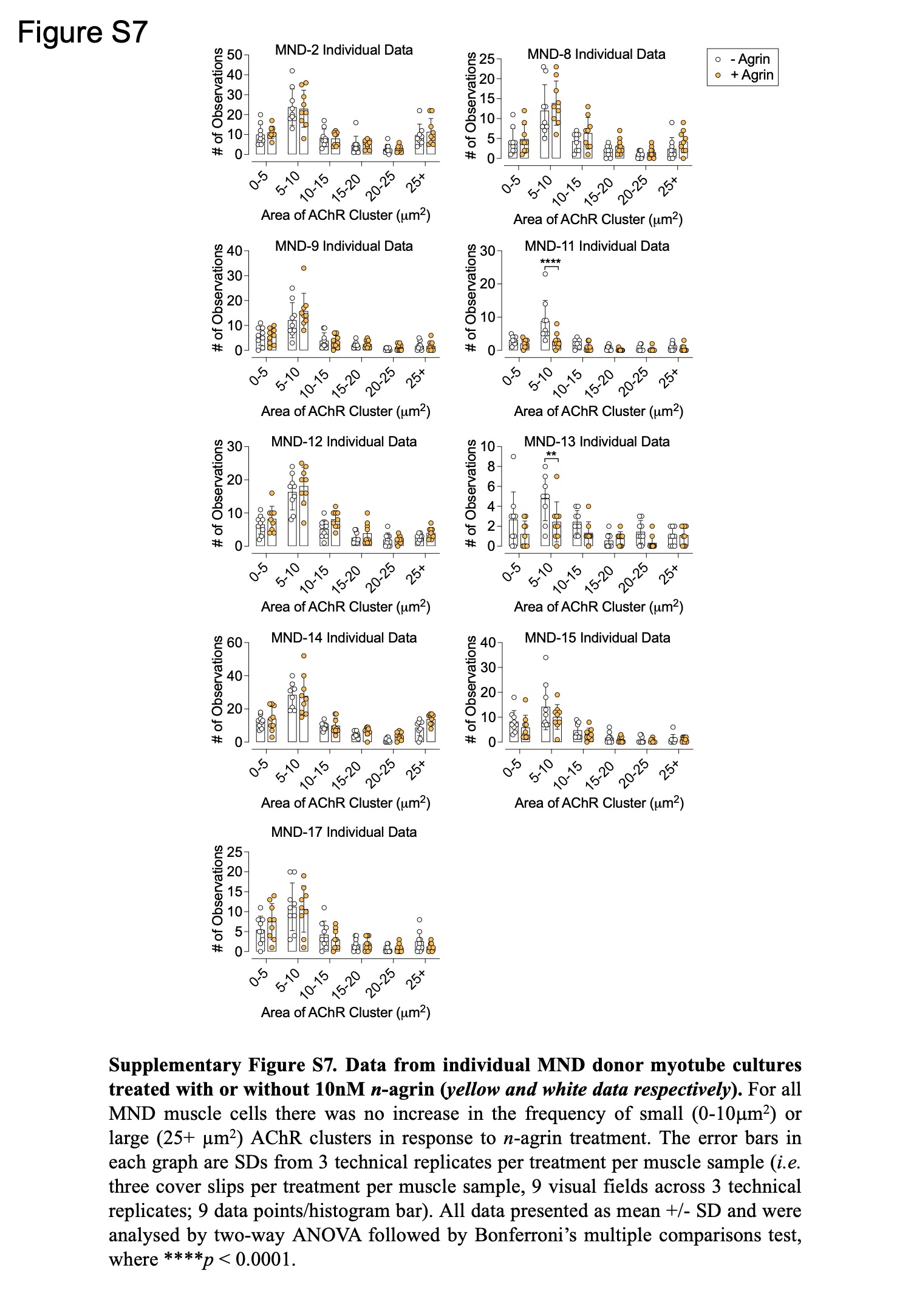
**
